# Transcriptomic landscape of prophase I sunflower male meiocytes

**DOI:** 10.3389/fpls.2014.00277

**Published:** 2014-06-16

**Authors:** Nathalia M. V. Flórez-Zapata, M. H. Reyes-Valdés, Fernando Hernandez-Godínez, Octavio Martínez

**Affiliations:** ^1^Laboratorio Nacional de Genómica para la Biodiversidad (Langebio), Centro de Investigación y de Estudios Avanzados del Instituto Politécnico Nacional(Cinvestav) Irapuato, México; ^2^Department of Plant Breeding, Universidad Autónoma Agraria Antonio NarroSaltillo, México

**Keywords:** meiosis, transcriptome, RNA-seq, meiocytes, sunflower, meiotic genes, HA89

## Abstract

Meiosis is a form of specialized cell division that generates gametes, allowing recombination of alleles and halving the chromosome number. Arabidopsis and maize are the plant models that have been most extensively studied to determine the genes involved in meiosis. Here we present an RNA-seq study in which gene expression in male meiocytes isolated during prophase I was compared to that in somatic tissues of the sunflower HA89 line. We sampled more than 490 million gene tags from these libraries, assembled them *de novo* into a sunflower transcriptome. We obtained expression data for 36,304 sunflower genes, of which 19,574 (54%) were differentially expressed (DE) between meiocytes and somatic tissue. We also determined the functional categories and metabolic pathways that are DE in these libraries. As expected, we found large differences between the meiotic and somatic transcriptomes, which is in accordance with previous studies in Arabidopsis and maize. Furthermore, most of the previously implicated meiotic genes were abundantly and DE in meiocytes and a large repertoire of transcription factors (TF) and genes related to silencing are expressed in the sunflower meiocytes. We detected TFs which appear to be exclusively expressed in meiocytes. Our results allow for a better understanding of the conservation and differences in the meiotic transcriptome of plants.

## 1. Introduction

Meiosis is a form of specialized cell division in which a single round of DNA replication is followed by two sequential cell divisions, leading to the formation of haploid gametes (Hamant et al., 2006). Meiosis increases genetic variation through the recombination of homologous chromosomes, which occurs during prophase I (Osman et al., 2011). Prophase I is the longest meiotic stage. In *A. thaliana*, for example, cells remain in this stage for approximately 30 of the 33 h required to complete meiosis in this plant (Armstrong et al., 2003). Prophase I is divided into five sub-stages: leptotene, zygotene, pachytene, diplotene, and diakinesis, in which the highly complex process of chromosome pairing, synapsis, and homologous recombination takes place (Pawlowski and Cande, 2005; Hamant et al., 2006). Previous studies have shown that the chiasma structure, a cytologically visible structure observed during homologous recombination, is also important for the correct chromosome segregation (Carpenter, 1994).

Understanding the molecular basis of homologous recombination may allow us to manipulate this mechanism with important repercussions for plant breeding efforts (Wijnker and de Jong, 2008; Wijnker et al., 2012). After decades of research, we now know that the regulation of early meiotic processes involves multiple levels of cellular organization, ranging from structural changes in the chromatin (Melamed-Bessudo and Levy, 2012), crossover balance (Phadnis et al., 2011), non-coding RNAs (Ding et al., 2012), protein degradation or phosphorylation (Sung and Klein, 2006; Shrivastav et al., 2008; Falk et al., 2010; Wu et al., 2010; Osman et al., 2011), and translational rate (Brar et al., 2012). In addition, transcriptional regulation of meiosis has been observed in yeast, in which expression of meiotic genes occurs at specific temporal windows (Vershon and Pierce, 2000; Mata et al., 2002) and some transcriptional regulatory cis-acting elements like *ABFI* control gene expression during meiosis (Ozsarac et al., 1997).

In lily plants (*Lilium longiflorum*), a GRAS-family protein designated as LISCL was proposed to act as a meiosis-associated gene expression regulator (Morohashi et al., 2003). Maize plants harboring different alleles of the *AMEIOTIC1* gene display differences in the transcriptional profile of meiotic genes in anthers, which suggests that this gene encodes a regulator of genes important for meiosis and prophase I progression (Pawlowski et al., 2009; Nan et al., 2011). The meiosis-associated protein MMD1 (Male Meiocyte Death 1), identified in *A. thaliana*, possesses a PHD domain that is often found in transcriptional regulatory proteins and proteins associated with chromatin-remodeling complexes, suggesting that this protein could act as a transcriptional regulator (Yang et al., 2003).

Transcriptome profiling of anthers in different plant species, such as wheat (Crismani et al., 2006), rice (Deveshwar et al., 2011), petunia (Cnudde et al., 2006), and Arabidopsis (Wijeratne et al., 2007) has resulted in the identification of conserved orthologs of meiosis-associated genes, new meiosis-related candidate genes as well as cellular pathways highly active in this plant structure. However, given that anthers contain both meiocytes as well as somatic cells, some of the genes and expression patterns described may not be meiosis-specific, and some important meiotic genes and processes could remain undetected due to the mixture of somatic and meiotic cell populations. The development of new methods for meiocyte collection, including the Capillary Collection Method (CCM) (Chen and Retzel, 2013), allowed for the description of the transcriptional landscape of Arabidopsis (Chen et al., 2010; Yang et al., 2011) and maize (Dukowic-Schulze et al., 2014a) meiocytes through RNA-seq. These studies detected high-level expression of transposable elements in Arabidopsis meiocytes (Chen et al., 2010; Yang et al., 2011), as well as many mitochondrial genes in both plant model systems (Chen et al., 2010; Dukowic-Schulze et al., 2014a). New meiosis-specific transcriptional regulatory elements were described (Li et al., 2012; Dukowic-Schulze et al., 2014a) and the similarities and differences in meiosis at the transcriptomic level between these species were discussed (Dukowic-Schulze et al., 2014a). Despite these advances, more research is needed to fully understand the similarities and differences in meiosis among plant species.

Sunflower (*Helianthus annuus* L.) is a multi-purpose crop (protein and vegetable oil source as well as ornamental flower), which is economically important (Seller and Jan, 2010) and has been used as a model organism for studies of diploid and polyploid hybrid speciation, introgression, and genetic architecture (Rieseberg et al., 1993, 1995). Whole-genome sequencing of this species is in progress (Kane et al., 2011). In addition, the technical feasibility of isolating nearly pure populations of male meiocytes in well-defined stages of meiosis without sophisticated techniques or laboratory equipment (Rodríguez-de-la Paz et al., 2007), makes this plant an excellent model for the study of meiotic gene expression. Here we present an RNA-seq transcriptome analysis of male sunflower meiocytes during prophase I, and compare it with a profile of a somatic library of tissues from the same line (HA89). We discuss commonalities and differences between our transcriptomes and previous reports of gene expression in meiocytes of Arabidopsis and maize.

## 2. Results

### 2.1. Overview of the transcriptome of sunflower meiocytes

**Figure 1:**
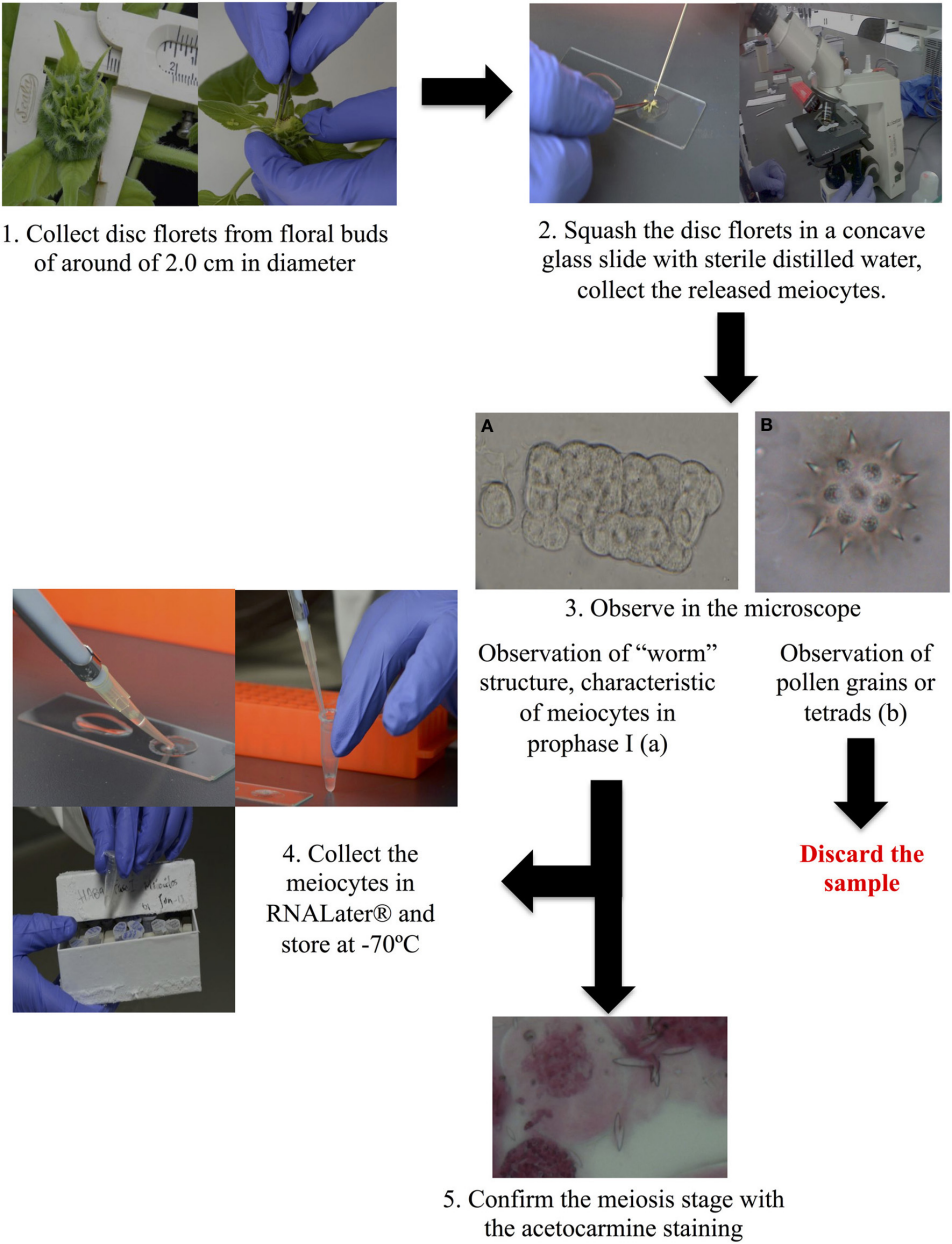
**Overview of the process of meiocyte isolation**.

With the aim of estimating and comparing the relative expression levels of genes in meiocytes and somatic tissues, three cDNA libraries were constructed and subjected to next-generation sequencing. Two libraries were derived from meiocytes that were carefully staged and collected at the prophase I stage (see Figure 1) and the third library was constructed from samples of RNA extracted from six somatic organs (leaf, stem, root, bract, corolla, and receptacle) and mixed in equimolar ratio. We obtained a total of 491,701,991 paired-end reads, which after trimming resulted in 387,767,222 (78.86%) high quality reads (see Methods and Table S1). These reads were assembled using a *de novo* transcriptome assembly approach, which yielded a total of 47,295 distinct transcripts (“components”). A total of 278,997,557 reads (71.94%) mapped to a unique locus within one of the transcripts, giving reliable evidence for the expression of 46,386 transcripts. In other words, 98% of all components had evidence of expression in these libraries. The remaining 2% of the components could not be matched to reads aligning uniquely within them and thus were excluded for downstream analyses. 32,303 (69.64%) of all the transcripts were annotated *via* a BLAST analysis resulting in at least one hit to a gene or protein in the databases queried (see Methods). The remaining 14,087 (30.36%) transcripts could not be identified using this approach. Identified transcripts that shared the same BLAST identifier were considered to be either products of the same sunflower locus or transcripts derived from closely related gene paralogs. To quantify the expression of these transcripts, reads aligning to these loci were added and the components were “collapsed” to treat the related components as a single gene. Our final dataset was composed of 36,304 genes which were used in the downstream analyses.

**Figure 2:**
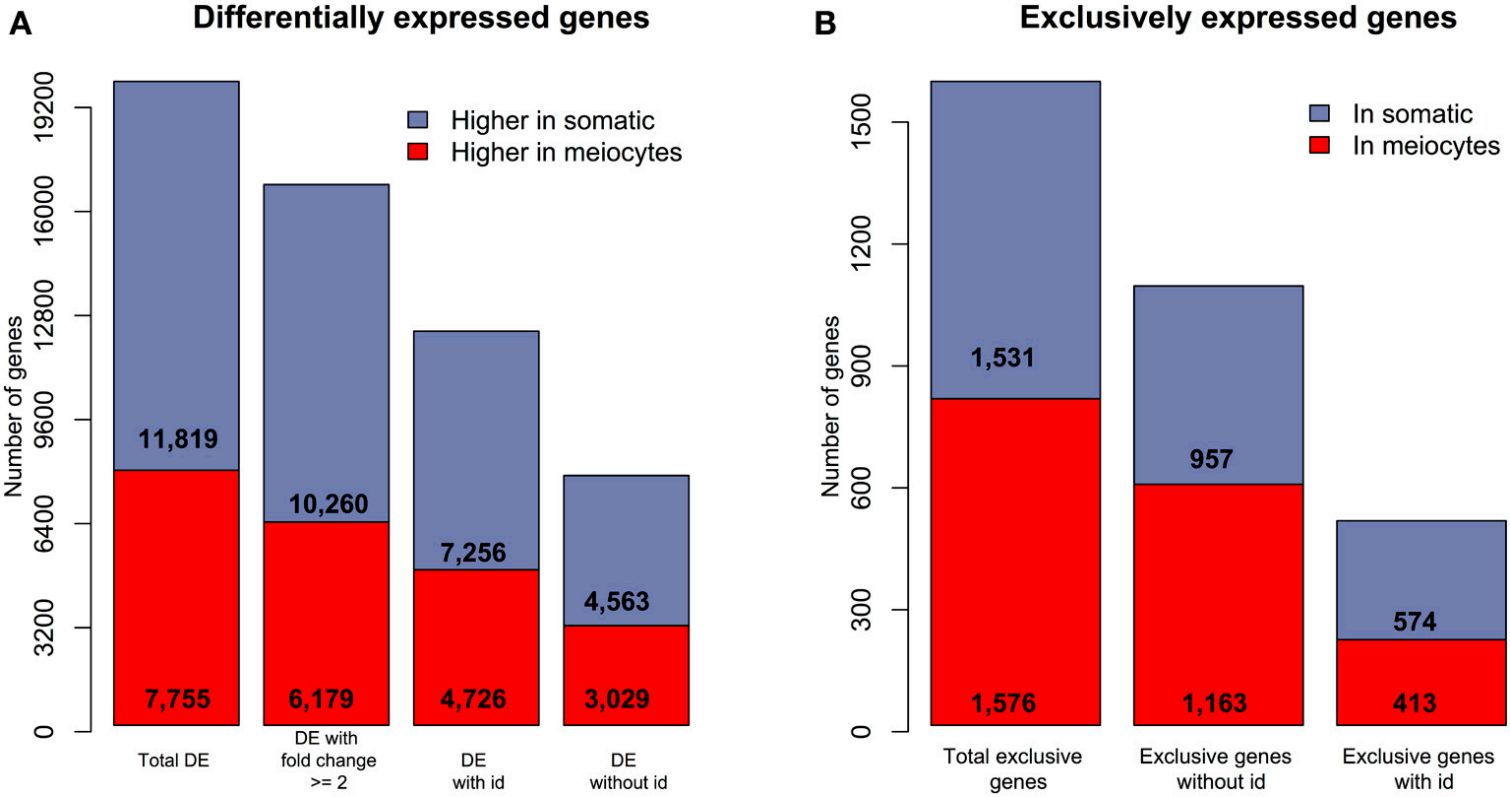
**Overview of differentially and exclusively expressed sunflower genes when comparing meiocytes and somatic libraries. (A)** The number of DE (FDR = 1%) genes in the two transcriptomes; “with id” are identified genes and “without id” are non-identified genes.**(B)** The number of genes with evidence of expression in only one of the two library types (“exclusive” genes).

A total of 19,574 (54%) genes were DE between meiocytes and somatic libraries using a False Discovery Rate (FDR) of 1%. Of these, 7,755 genes showed higher expression in meiocytes and 11,819 showed higher expression in somatic tissues. Approximately 80% of the DE genes showed a ≥2-fold change between the samples. Interestingly, 38.37 and 39.05% of the DE genes exhibiting higher expression in somatic tissues and meiocytes, respectively, were genes that could not be identified using our BLAST approach (Figure 2A). A subset of genes showed evidence for expression (at least one aligned read) in only one of the two types of libraries (meiocytes or somatic). Most of these genes were also non-identified (genes without id; Figure 2B).

We identified 63 orthologs from a total of 84 previously described *A. thaliana* genes (Chen et al., 2010; Yang et al., 2011) that are associated with meiosis. Of this subset of known meiotic genes, 51 were found to be DE, and only two of them exhibited higher expression in somatic tissues (Figure 5). Furthermore, we found that MMD1, a gene that is important for male meiosis, was the one with the greatest difference in gene expression (around of 1000 times higher in meiocytes that in somatic tissues), which confirms the reliability of our data. Also, we observed that all of the DE genes that showed higher expression in meiocytes had greater than a ≥2-fold-change relative to expression in the somatic tissues.

### 2.2. Functional categories and metabolic pathways up-regulated in meiocytes

We categorized the sunflower gene orthologs into Gene Ontology (GO) functional categories including Cellular Components (CC), Biological Processes (BP), and Molecular Functions (MF) in order to describe biological differences between the somatic and meiocyte transcriptomes. Using the methodology previously reported in Martínez-López et al. (2014) we found 160 CC GO terms (from a total of 189 considered), of which 40 were over-represented by at least ≥2-fold in meiocytes (Table S2); as expected, within these terms are those related to cell division and chromatin organization, such as “condensin complex,” “kinetochore,” “chromatin assembly complex,” “nucleosome,” “microtubule associated complex.” as well as terms related to transcription and mitochondria [“mitochondrial outer membrane translocase complex,” “mitochondrial intermembrane space,” and “mitochondrial proton-transporting ATP synthase complex, coupling factor F(o)”]. In the somatic transcriptome we observed a higher representation of terms related to CC in which photosynthesis takes place (Table S3).

**Figure 3:**
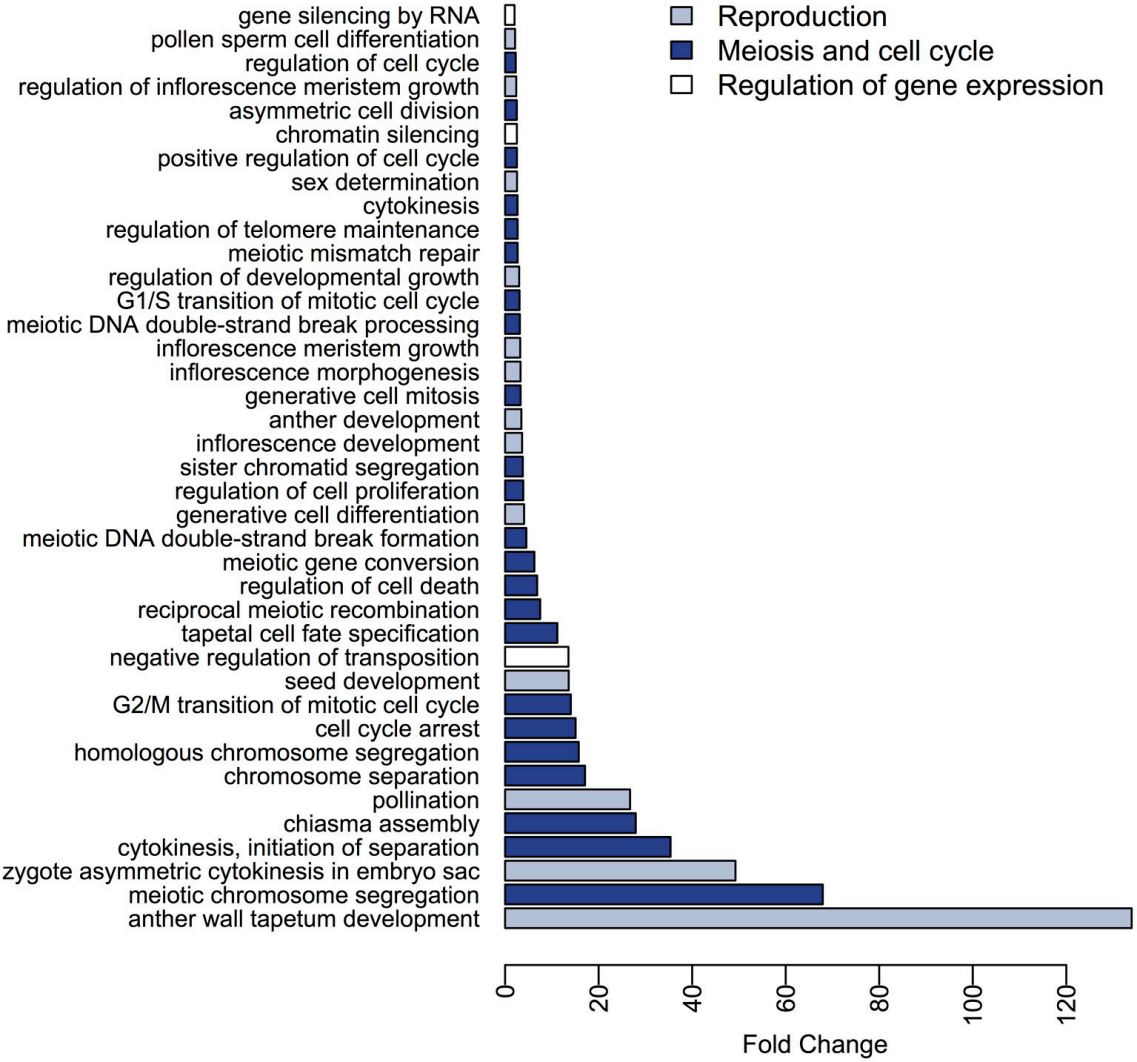
**Differences in expression of BP GO terms between the meiocytes and somatic transcriptomes**. The BP terms with higher expression in meiocytes and a fold-change ≥ 2 are presented.

Our sunflower gene dataset was grouped into 386 BP GO terms, and 338 of these were found to be differentially represented between the meiocytes and somatic transcriptomes. Among the terms that showed a ≥2-fold change in meiocytes (79 BP GO terms), are terms related with reproduction such as “pollination,” “pollen sperm cell differentiation,” and “sex determination” as well as terms associated with meiosis and cell cycle including “meiotic chromosome segregation,” “chiasma assembly,” “reciprocal meiotic recombination,” “meiotic DNA double-strand break formation,” and categories related to gene silencing and regulation of gene expression as “negative regulation of transposition,” “chromatin silencing,” “gene silencing by RNA” (Figure 3, Table S4). On the other hand, a wide range of BP GO terms were found with significantly higher expression in somatic tissues when compared with meiocytes. Several of these terms were related to “response to stimulus” and “photosynthesis” (Table S5).

When we explored the differences in GO terms participating in the MF category (151 terms were considered in this category), we found that, although 139 were DE, only 8 had greater than a ≥2-fold expression change in meiocytes (Table S6). Of these, “histone binding,” “cyclin binding,” “nucleosome binding,” and “ATPase activator activity” are related to the CC and BP categories found with higher expression in meiocytes. In a similar manner, the terms highly expressed in the somatic tissues in this category are related to those found more highly expressed in the CC and BP categories for this transcriptome, e.g., “ribulose-1,5-bisphosphate carboxylase/oxygenase activator activity” and “chlorophyll binding” (Table S7).

**Figure 4:**
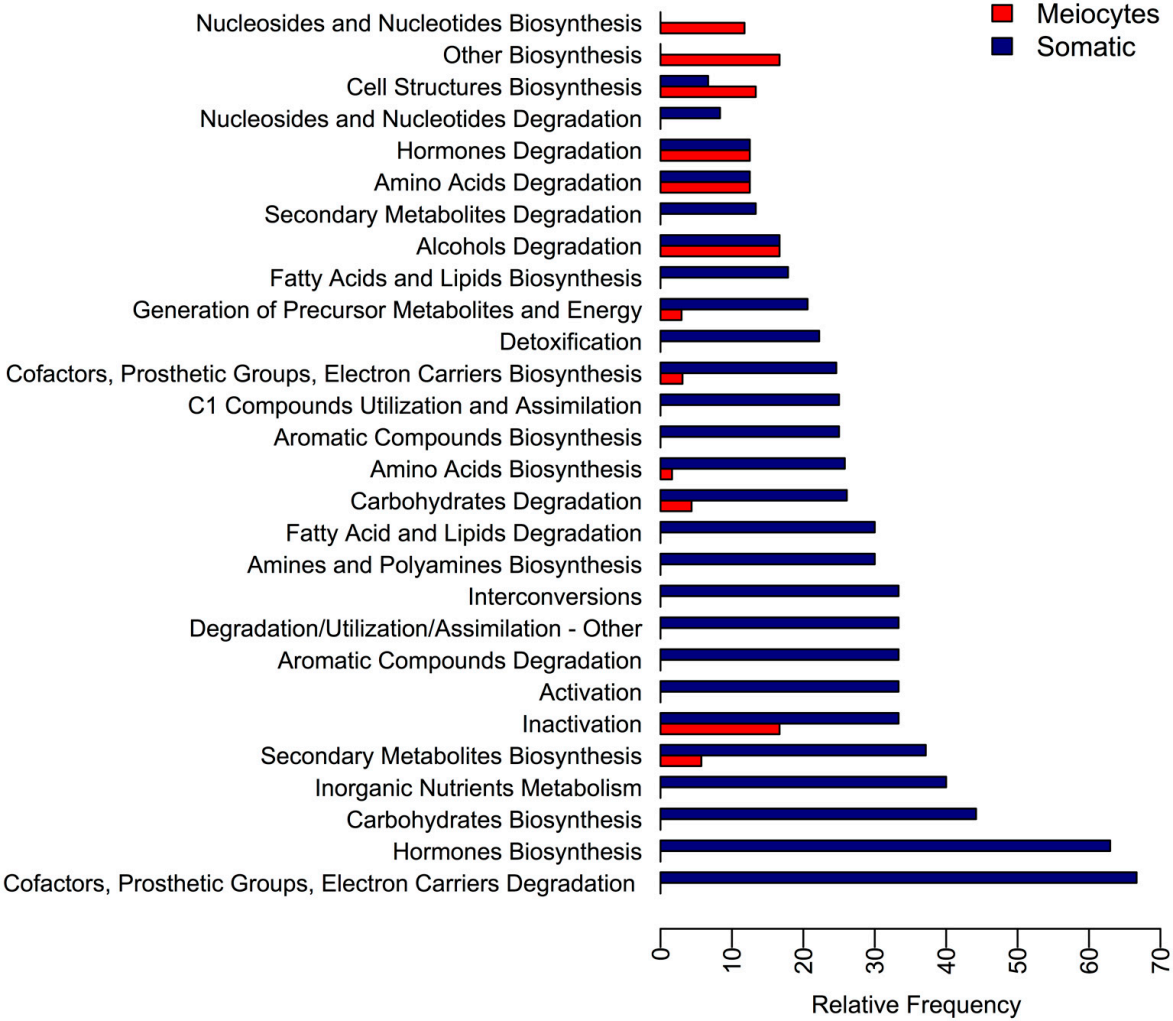
**Relative frequency of metabolic pathways found differentially expressed at each metabolic pathway hierarchy**. The DE pathways were grouped into their hierarchy category and the relative frequency at which the pathway of each category were found DE is presented.

Another approach to explore the functions of genes present in our data was to describe the differences in the transcriptomes in terms of metabolic pathways. With this aim we categorized the sunflower gene dataset into 370 distinct metabolic pathways of which 323 were found to be DE and 22 showed greater than a ≥2-fold increase in meiocytes (Table S8) while 167 showed greater than a ≥2-fold increase in somatic tissues (Table S9). Moreover, we took advantage of the previously established Aracyc pathway hierarchy and calculated the relative frequency at which the pathways belonging to a particular hierarchy were DE with a fold-change greater than 2 (Figure 4). From this analysis we concluded that most of the metabolic pathways showed higher expression in somatic tissues, with the exception of pathways in the “Cell Structures Biosynthesis,” “Nucleosides and Nucleotides Biosynthesis,” and “Other Biosynthesis” hierarchies, which were more highly represented in meiocytes.

**Figure 5:**
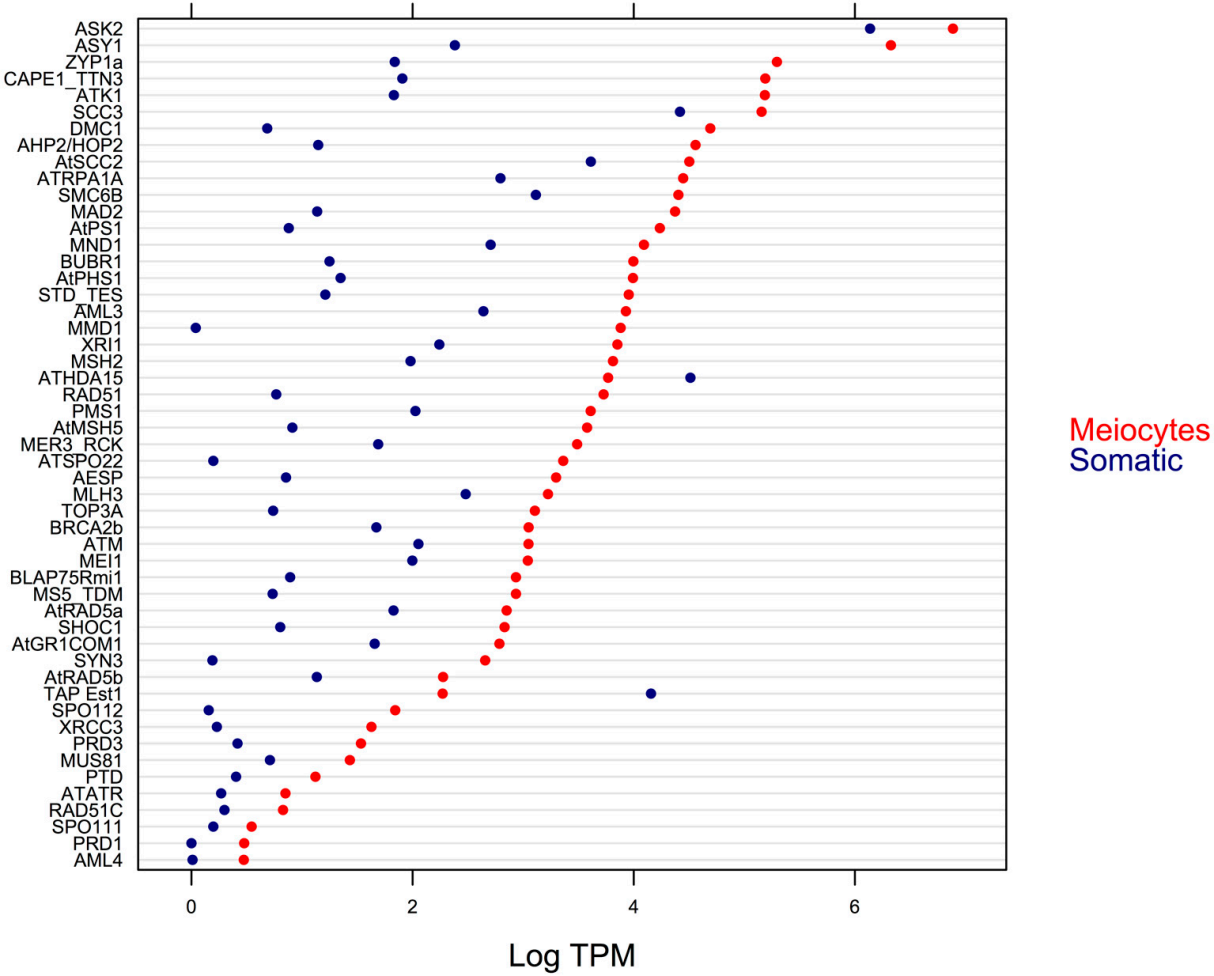
**Expression level (in logarithm of the TPM) of DE genes with previously established function in meiosis**.

In addition to identifying putative sunflower orthologs of 63 of the 84 meiotic genes previously described in Arabidopsis Chen et al. (2010); Yang et al. (2011), we attempted to determine if the remaining 21 meiosis-associated genes are conserved in other plant species (see Figure S1). For this set of 21 genes not identified in our sunflower transcriptomes, we searched for orthologs in 25 plants in the Plaza v 2.5 database (Proost et al., 2009). The number of orthologs for these 21 genes, as well as their distribution among plant species greatly varies. For example the *ATRSP3* (AT1G64030) gene appears to be conserved only in the Arabidopsis genus, while the *AHP2* (AT3G29350) gene, which has orthologs in most of the species studied, could not be detected in the *Oryza* genus. Thus, it is not surprising that we did not detect the expression of some meiosis-associated genes in our meiocyte transcriptome. On one hand, these genes may not be present in the sunflower genome or their level of expression was below a detection threshold. Alternatively, gene sequences may have diverged significantly such that they could not be identified as orthologs.

### 2.3. Expression levels of transcription factors

In order to identify transcriptional regulators preferentially or exclusively expressed in meiocytes, we analyzed the expression pattern of known TF genes in our dataset. We found 489 DE TF, 188 with higher expression in meiocytes and 301 with higher expression in somatic tissues. These TF belong to 54 different families (Jin et al., 2014) (Figure S2). We found a larger number of distinct TF families that showed higher expression in meiocytes, compared to those observed with higher expression in somatic tissues (49 vs. 38 TF families, respectively). In addition, we found 14 TF whose expression was detected only in meiocytes (Table S10). The distribution of the number of TF per family was significantly different between meiocytes and somatic libraries (*P* ≈ 6.1*e* − 13).

Gene silencing by RNA was one of the GO BP with significantly higher representation in meiocytes. We observed differences in the expression of 36 genes that were previously associated with RNA-regulated gene silencing pathways (Table S11). We found 29 orthologs of these genes in our sunflower dataset. Eighteen genes were DE between the meiocytes and somatic tissues, and, of these, 17 genes were DE greater than ≥2-fold in meiocytes compared to somatic tissues (Figure S3).

### 2.4. qRT-PCR analysis of selected genes

**Figure 6:**
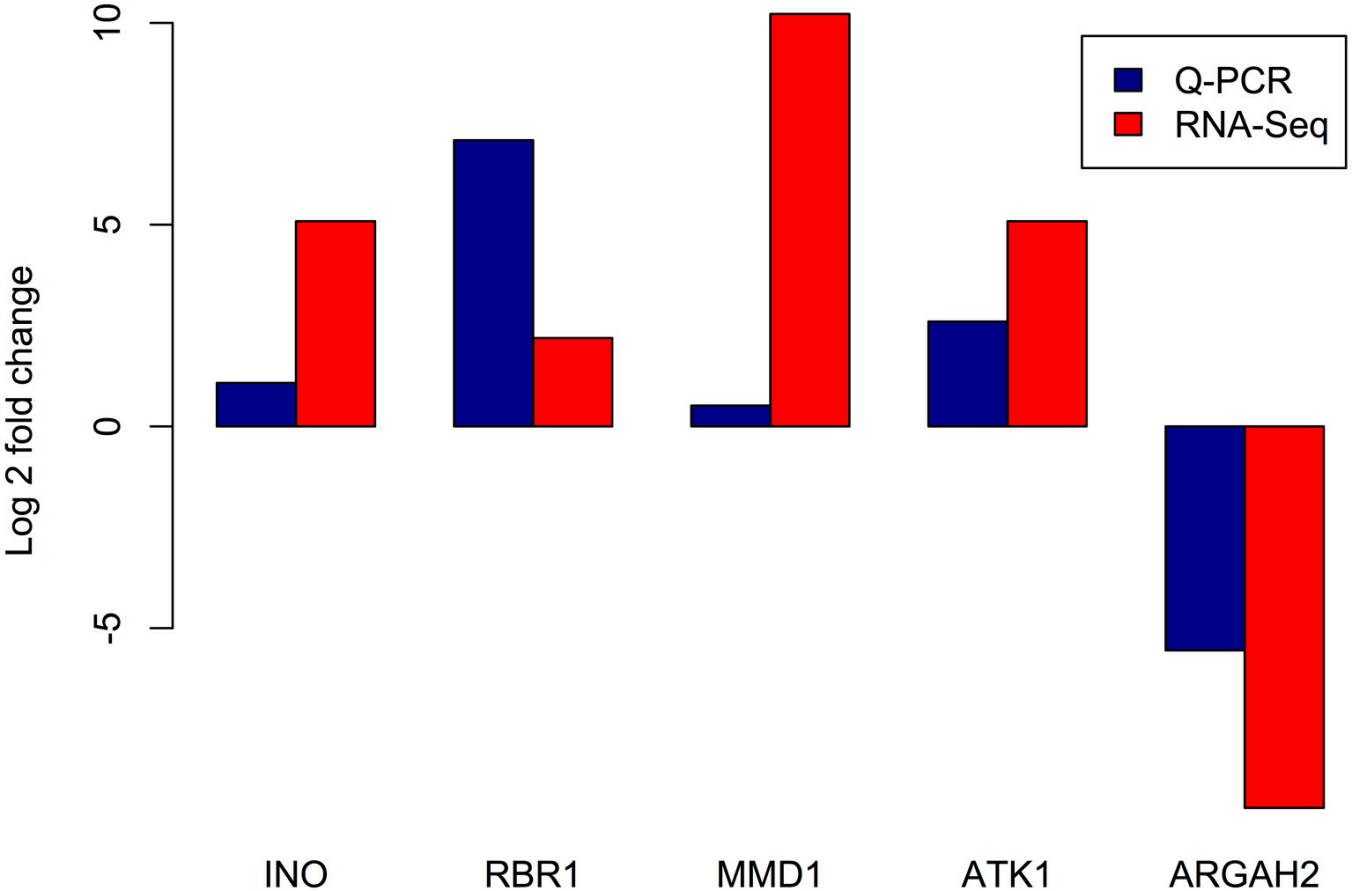
**Comparison of expression levels of selected genes estimated by RNA-seq and RT Q-PCR**. X axis: tested genes, *INO* - “*INNER NO OUTER*” transcription factor, *RBR1* - *RETINOBLASTOMA RELATED1*, *MMD1* - *MALE MEIOCYTE DEATH1*, *ATK1* - *ARABIDOPSIS THALIANA KINESIN1*, and *ARGAH2* - *ARGININE AMIDOHYDROLASE2*. Y axis - log_2_ of estimated fold change between meiocyte and somatic libraries.

In order to validate the gene expression levels detected in our RNA-seq experiment, we used an independent method and estimated the expression of five genes by qRT-PCR. According to the RNA-seq data, four of these genes exhibited higher expression in meiocytes and the fifth showed higher expression in the somatic library. Genes exhibiting higher estimated expression in meiocytes were: *INO*, encoding the “*INNER NO OUTER*” TF which showed relatively low expression (7.42 TPM in meiocytes and <1 TPM in somatic tissues) and was previously related with ovule development (Baker et al., 1997); *RBR1*, encoding a retinoblastoma related protein, and is essential for early meiotic events in Arabidopsis (Chen et al., 2011); Two well-studied meiotic genes, *MMD1* (*MALE MEIOCYTE DEATH1*) and *ATK1*, encoding a kinesin required for spindle morphogenesis in male meiosis (Chen et al., 2002). As an example of a gene with higher average expression in the somatic library, we selected *ARGAH2* (encoding arginine amidohydrolase 2) which is implicated in pathogen defense (Gravot et al., 2012). Expression levels of these five genes as estimated by both RNA-seq and qRT-PCR are presented in Figure 6. Even when, as expected, fold changes are highly variable between methods (Marioni et al., 2008), for each of the five genes, the differences in expression are consistent between RNA-seq and qRT-PCR.

## 3. Discussion

### 3.1. The meiocyte transcriptome is distinct from the somatic transcriptome in sunflower

It is important to note that merging somatic tissues in a single source of RNA (the somatic library) allowed us to make a direct comparisons between meiocytes and “somatic” gene expression. However, this procedure does not guarantee that a gene found to be DE between the meiocyte and somatic libraries would be DE between meiocytes and any one of the individual tissues that comprise the somatic tissue pool. In other words, our comparisons between meiocytes and “somatic” gene expression levels are only valid for the “average” somatic expression estimated in the particular library constructed.

Differential expression analysis of the sunflower male meiocyte transcriptome against its somatic counterpart revealed that most of the DE genes showed higher expression in somatic tissues in comparison to meiocytes. This observation is likely due to the fact that the somatic transcriptome is derived from a mixture of different plant tissues, which require a wide range of biological functions. It is reasonable to assume that they require the expression of many genes, while meiocytes are a specialized cell type that have, as their primary function, to produce the haploid gametes through meiosis. This cell specialization may explain why a subset of genes was characterized by expression in only the somatic or meiocyte libraries, but not both. Interestingly, the number of library-specific genes that could not be annotated by similarity in a BLAST search was slightly higher in meiocytes in comparison to the somatic tissues. In Arabidopsis and maize meiocyte transcriptomes, this bias toward the detection of un-annotated features was also observed (Dukowic-Schulze et al., 2014a), suggesting that many of the genes that confer meiocyte cell identity remain uncharacterized.

### 3.2. Meiosis related functions are the predominant functional feature in the sunflower meiocyte transcriptome

We employed the methodology described in Martínez-López et al. (2014) to analyze the statistical significance of GO functional categories. These categories include the sunflower genes that could be identified as orthologs of Arabidopsis genes participating in these ontologies. From this analysis, we determined that most of the terms in the BP category with higher expression in meiocytes were terms related to reproduction, meiosis and cell division, which is in agreement with Dukowic-Schulze et al. (2014a). That group also found that these terms are up-regulated in the early meiosis transcriptome of Arabidopsis. However, in their study, up-regulated terms in the maize transcriptome were mostly related to metabolic and biosynthetic processes, and the authors argued that some of these differences could be due to poor maize gene annotation. In this work we used Arabidopsis annotation by ortholog identification as a reference for our functional analyses, obtaining results that are consistent with the function and specificity of meiocytes.

Terms DE in the CC and MF categories between meiocyte and somatic transcriptomes also pointed to functions related to the energetic activity of meiocytes, like “ATPase activator activity,” “mitochondrial intermembrane space” and “mitochondrial outer membrane translocase complex,” among others. Again, this is consistent with previous reports on the meiocyte transcriptomes of maize and Arabidopsis (Chen et al., 2010; Dukowic-Schulze et al., 2014a), in which genes important for mitochondrial function were up-regulated in meiocytes. Other studies discuss that proteins required for homologous recombination, such as Rad50, DMC1, and Rad51 have an ATPase dependent activity (Hopfner et al., 2000; Nara et al., 2001), reinforcing the importance of energy metabolism in these specialized cells.

When we analyzed the differences between metabolic pathways represented by genes expressed in the meiocyte and somatic transcriptomes, we found that most categories showed higher expression in somatic tissues. However, three metabolic pathway hierarchies exhibited higher expression in meiocytes. One of these was “nucleosides and nucleotides biosynthesis” and, according to previous studies in yeast, synthesis of macromolecules including nucleotides are observed as a component of metabolic changes that occur during the transition to mitosis from meiosis (Ray and Ye, 2013). In addition, Moss et al. (2010) established that DNA damage-induced nucleotide synthesis is important in homologous recombination DNA repair in yeast. Downs (1997) showed that purine nucleotide-generating pathways are involved in the regulation of hormone-induced meiotic maturation in mouse oocytes. “Cell structures biosynthesis” was the second pathway hierarchy found to be highly expressed in meiocytes. This pathway is of particular interest because the “sporopollenin precursor biosynthesis” sub-hierarchy exhibited the largest difference in expression between meiocytes and somatic tissues. Sporopollenin is the major component of the exime, the outer pollen wall (Domínguez and Mercado, 1999) and thus this is not an unexpected result. Finally, “PRPP biosynthesis I” is one of the metabolic pathways that belongs to the “other biosynthesis” pathway hierarchies that were found to be more represented in meiocytes. Phosphoribosyl pyrophosphate (PRPP) is a pentosephosphate which was previously implicated in meiotic induction in mouse oocytes (Downs et al., 1998).

### 3.3. The majority of genes previously implicated in meiosis are preferentially and highly expressed in the sunflower meiocyte transcriptome

A set of 84 genes has been described as meiosis-associated in Arabidopsis (Chen et al., 2010; Yang et al., 2011). Of these, we were able to identify 63 sunflower putative orthologs in our transcriptomes. In maize, orthologs for 65 of the 84 Arabidopsis meiotic genes were found, even when a total of 82 paralogs are reported (Dukowic-Schulze et al., 2014b). When we explored the conservation of gene families within which known meiosis-related genes could not be identified in our sunflower data, we observed that, with the exception of *MRE11* and *ATK5*, these genes do not appear to be conserved in plants. The number of genes that belong to each family also varies between plant species (see Figure S1).

For eight of the 21 meiotic genes whose expression was not detected in our transcriptomes (*ZYPb, ASK1, AML2, BUB3.1, ATSMC2, BRCA2A, ATK5*, and *SMC6A*), we detected expression of at least one sunflower ortholog belonging to the same family. This result suggests that, although we cannot exclude technical challenges in detecting weakly expressed genes or the difficulty of finding the ortholog of these meiotic genes, it is possible that orthologs of these genes are absent in sunflower or the number of paralogous genes participating in these genes families are different from those reported in Arabidopsis. Analyses at the genomic level in sunflower are needed to confirm this observation.

Differential expression for 51 of the 82 known meiotic genes was observed, and only two of these genes exhibited higher expression in the somatic tissues (encoding the telomerase-activating protein Est1 and the ATHDA15 histone deacetylase, which correspond to AT1G28260 and AT3G18520 loci, respectively). According to Alinsug et al. (2009), the histone deacetylase ATHDA15 is significantly expressed at the root tip and pollen, whereas Liu et al. (2013) found that this histone deacetylase is a negative transcriptional regulator of genes important for chlorophyll biosynthesis and photosynthesis in etiolated seedlings. Together, these results confirm that this gene plays a role in non-meiotic cells, thus its higher expression in somatic tissue could be expected. The telomerase-activating protein Est1 is an Arabidopsis ortholog of the SMG7 human protein, which has a role in nonsense-mediated RNA decay (Riehs et al., 2008). This gene was up-regulated in response to root flooding in Arabidopsis (Hsu et al., 2011), suggesting a non-exclusive meiocyte expression.

A total of 12 (19.04%) sunflower orthologs of known meiotic genes in Arabidopsis were not DE between somatic and meiocyte transcriptomes. This result is in agreement with expression data for these genes in Arabidopsis and maize. In Arabidopsis, only 72.05% of the meiotic genes presented greater than a ≥2-fold difference in expression when comparing meiocytes with seedlings (Chen et al., 2010) and in maize, only 57.31% of the genes had greater than a ≥2-fold change in meiocytes compared with seedlings (Dukowic-Schulze et al., 2014b). Among the meiotic genes not DE in sunflower, we found that *ZIP4, MPA1, CDC45* and *SMC1_TTN8* orthologs were also not more abundantly expressed in maize meiocytes (Dukowic-Schulze et al., 2014b). Other genes with important roles in meiosis also have functions in mitosis or in DNA mismatch repair, such as *MLH1, MSH4, RAD50*, and *AML5* (Chen et al., 2010; Osman et al., 2011; Yang et al., 2011). Other genes such as *SMG7* exhibit a broad tissue distribution in their expression (Riehs et al., 2008). Results from our study highlight that these types of genes do not show a significant change of expression when comparing meiocytes with somatic tissues.

### 3.4. A large repertoire of transcription factors (TF) and genes related to silencing are expressed in the sunflower meiocytes

Our results suggest that a larger variety of TF families are expressed in meiocytes when compared with somatic tissues; we found that 16 families were expressed only in meiocytes. In our data, bHLH and MYB TF families were two of the most expressed in meiocytes, which is in agreement with the observations of Dukowic-Schulze et al. (2014a) of maize and Arabidopsis meiocytes. However, these families were also highly represented in somatic tissues. TFs belonging to the C2H2 family showed the second highest expression differences in meiocytes and also higher expression compared with somatic tissues. Interestingly, in *C. elegans*, four related C2H2 zinc-finger proteins play a central role in homologous chromosome pairing and synapsis during meiosis (Phillips and Dernburg, 2006). In Drosophila, a C2H2-type zinc finger TF (Grauzone) is required for the completion of meiosis in oocytes (Chen et al., 2000). We found that 8 TFs of the GATA family were highly expressed in meiocytes compared with somatic tissues. Recently a GATA Factor, Gaf1, was described as a regulator of sexual development (mitotic to meiotic transition) in yeast *Kim2012*, suggesting that TFs of this type could also play an important role during meiosis. The FAR1 family of TFs was expressed only in meiocytes, and is also the one of the set with higher expression in meiocytes. Members of this TF family are proteins related to the mutator-like transposases *Lin2007*. Further studies are needed to establish if this finding pertains to previous observations of high activity of transposable elements in Arabidopsis meiocytes (Yang et al., 2011; Chen and Retzel, 2013).

In comparison to the set of TFs up-regulated in meiocytes in both Arabidopsis and maize described in Dukowic-Schulze et al. (2014a), we did not detect evidence for the expression of six sunflower orthologs in our transcriptomes (Arabidopsis locus identifiers - AT1G02030, AT1G06170, AT2G36270, AT2G42830, AT5G17800, and AT5G19790). Furthermore, an additional three TF orthologs had no evidence of differential expression between the meiocytes and somatic tissues (AT3G54340, AT5G20240, AT5G60910). Several TF consistently up-regulated in meiocytes are TF involved in flower development such us CRAB CLAW, AMS and AGAMOUS (Dukowic-Schulze et al., 2014a). In all three plant species, an additional two conserved TF showing higher expression in meiocytes are not annotated and could be interesting genes for further study of the regulation of transcription in meiocytes. This analysis of sunflower TF expression in the meiocyte transcriptome suggests the activity of diverse transcriptional regulators during meiosis, as proposed in Yang et al. (2011).

Finally, we explored whether the expression of genes involved in RNA-regulated gene silencing pathways was higher in meiocytes than in somatic tissues as suggested by our BP GO analysis. We found orthologs for 29 distinct genes with this role and, of these, 18 were DE between somatic tissues and meiocytes. Seventeen genes showed greater than a ≥2-fold increase in expression in meiocytes. This machinery is clearly important for silencing of transposons (Eamens et al., 2008), and as proposed by Yang et al. (2011), the higher activity of these silencing pathways could be a defensive mechanism in meiocytes to prevent mutations that could result by the movement of these elements in the germline. Further studies are needed in order to define the effects of silencing pathways and transposons on the regulation of meiotic gene expression.

### 3.5. Conclusions

This work allowed us to identify similarities and differences between the genes and mechanisms involved in meiosis in sunflower, in comparison to previous findings in Arabidopsis and maize. As expected, most of the key meiotic genes are conserved among the three species. However, we found many genes that could not be annotated in sunflower that are differentially or exclusively expressed in meiocytes. This suggests that many of the genes that confer meiocyte cell identity remain uncharacterized.

## 4. Materials and methods

### 4.1. Meiocytes collection, RNA extraction, and sequencing

Sunflower plants from the inbred line HA89 were grown under greenhouse conditions, in 14 *dm*^3^ pots containing a mix of leaf litter, sand, clay, and perlite (2:1:1:1). Plants were fertilized weekly with the Long Ashton nutrient solution (Phillips and Jennings, 1976) until the beginning of the R2 development stage (Schneiter and Miller, 1981). At this stage, when the floral buds were around of 2.0 cm in diameter [early meiotic stages, according to our previous observations as well as (Rodríguez-de-la Paz et al., 2007)] approximately 10 disc florets of the floral bud were harvested and placed on a concave glass slide with 80μL of sterile distilled water, and squashed with dissecting needles to release the meiocytes. Collected meiocytes were observed by microscopy without staining in order to verify that they remained associated with each other, forming the “worm” structure characteristic of meiocytes in prophase I (Yang et al., 2011) (see Figure 1). If tetrads or pollen grains were observed, the floral buds were discarded. This protocol is a modification of one previously reported in Rodríguez-de-la Paz et al. (2007). Meiocytes from floral buds that were in prophase I were collected in RNAlater solution (Qiagen, Valencia, CA) and stored at −70°C until RNA extraction. A developmentally-matched subset of the remaining disc florets were fixed with an ethanol 96%: acetic acid solution (3:1) for 24 h. In these fixed samples, meiocytes were observed using the squashed-acetocarmine staining method to confirm the meiotic phase of the meiocytes. In florets containing pure populations of prophase I meiocytes, the frozen, matched sample was used for RNA extraction.

Total RNA of meiocytes confirmed to be in prophase I was isolated using the ZR RNA MicroPrep kit (Zymo Research, Orange, CA) following the manufacture instructions, and stored at −70°C. RNA from somatic tissues (leaf, stem, root, bract, corolla, and receptacle) was extracted with the NucleoSpin RNA Plant kit (Macherey-Nagel, Düren, Germany) and mixed in equimolar amounts. This mixture was used later for the preparation of the somatic library. Two libraries from meiocytes (biological replicates) and one library of somatic tissues were prepared using standard Illumina TruSeq RNA library kits, and sequenced using the Illumina HiSeq 2500 platform to obtain 100 bp paired-end reads.

### 4.2. Quality filtering, *de novo* assembly, and remapping

After sequencing, adaptors were removed from the reads using “cutadapt 1.3” software (Martin, 2011), with the sequences reported by Illumina for the TruSeq DNA v1/v2/LT, RNA v1/v2/LT, ChIP Sample Prep Kits, and Paired End DNA oligonucleotides. Reads were then quality trimmed using PRINSEQ 0.20.4 software (Schmieder and Edwards, 2011), allowing a minimum quality score of 20 and no more than two ambiguous bases per read. *De novo* assembly of the trimmed reads was performed using Trinity (release 20130225) software (Grabherr et al., 2011) using default parameters, with the exception of –min_kmer_cov that was set to two. The Trinity assembler outputs a set of “components” which correspond to sequences that the algorithm considers are product of different genes; each component is represented by one or more sequences that are putative splice variations of the transcripts. In order to assess relative gene expression levels, the quality-filtered reads were remapped to the representative components of the assembled sequences (genes) using Bowtie2 2.1.0 (Langmead and Salzberg, 2012) with parameters -a –rdg 6, 5 –rfg 6, 5 –score-min L, -0.6, -0.4. The unique read counts for each component, i.e., those reads that mapped exclusively to one component, were estimated using eXpress 1.4.1 (Roberts and Pachter, 2013) with default parameters. These counts were arranged in a matrix for downstream analysis.

### 4.3. Sequence identification

The components of the assembled transcriptome were identified by the best BLASTx hit [NCBI BLAST, (Altschul et al., 1997)] against four different databases: *A. thaliana* (TAIR10) peptides BLAST dataset, RefSeq plants peptides (release 24/07/2013), and sunflower peptides dataset for the varieties Ha412 and HaXRQ, available in the HeliaOrg website (https://www.heliagene.org). Only hits with a bit score of ≥90 and below an expected value *E* ≤ 1 × 10^6^ were considered as significant. If no hit fulfilling these criteria were found, then the sunflower transcript was classified as “non-identified” (see Table S2). The annotation of genes with GO terms (Harris et al., 2004) and Aracyc metabolic pathways (Mueller et al., 2003) were conducted using the R packages org.At.tair.db and GO.db (Pages et al., 2008). For TF identification, the Plant TFDB v. 3.0 (Jin et al., 2014) was used to extract the *A. thaliana* TF information. All data resulting from sequencing, assembly and annotation procedures were collected into a MySQL relational database (Server version 5.5.34).

### 4.4. qRT-PCR reactions

For qRT-PCR analysis, new RNA extractions were prepared from isolated prophase I meiocytes (around of five floral buds from different plants were used), also a new RNA equimolar mixture of somatic tissues (from a single plant) were prepared.

cDNA was obtained from total RNA (1.5 ug per reaction) with the High capacity RNA-to-cDNA kit (Applied Biosystems, Foster City, CA). Real- time PCRs were then carried out in 20 μL reaction mixtures containing 10 μL of 2 × SYBR Green PCR Master Mix from Applied Biosystems, 0.4 μL of each primer (10 μmol), 1.5 μL of cDNA (150 ng/μL), and 7.7 μL of distilled water. The PCR program was run in a StepOne instrument (Applied Biosystems, Foster City, CA), as follows: an initial denaturation for 10 min at 95°C, followed by 40 cycles of 95°C for 15 s and 1 min at 58°C. Three technical replicates were performed for each gene quantification. Melting curve analysis was conducted following PCR to verify that a single product was amplified. Relative differences in gene expression were detected via the ΔΔ*Ct* method using the ribosomal S5 gene as a loading control.

### 4.5. Statistical design and analyses

We followed the principles recommended in Auer and Doerge (2010) to obtain valid inferences in RNA-seq studies by including two biological replicates of the meiocyte libraries. For each one of the two libraries we used different sets of plants (floral buds, florets), performed independent total RNA isolation as well as library construction and sequencing. Variation in expression found for each gene between the two biological replicates gives an estimate of the statistical error (unexplained variation) which includes biological as well as technical variation. We did not obtain replicates from the somatic library and thus we assume that the statistical error within the estimates in the somatic library is of the same size as the one detected between replicates in meiocytes. Even when the level of replication is the minimum possible, for example only two replicates in one of the two treatments, the inference is still valid given that we have a source to estimate the error. As demonstrated in Robinson (2008) the estimation of the negative binomial dispersion is efficient even with small number of replicates. Furthermore, the statistical tests employed via the edgeR package (Robinson et al., 2010) are moderated statistical tests for assessing differences in tag abundance (Robinson and Smyth, 2007). This method employs estimates of global as well as gene-specific dispersion and is reliable for small samples.

Statistical analyses were performed in R Core Team (2013) version 2.15.3 (2013-03-01). As described above, the expression level of the components was considered as the unique read counts obtained for each one. However, for components sharing the same TAIR10 identifier, expression data were collapsed to a single component by summing the numbers of reads that mapped to each component. Differential gene expression analysis between meiocytes and somatic libraries was performed in the R package edgeR (Robinson et al., 2010), by using the exactTest function. The resulting *p*-values were input into the *q*-value function (Storey and Tibshirani, 2003) with default parameters, setting fdr.level = 0.01 to obtain a FDR of 1%. Differences in the GO functional categories, as well as in metabolic pathways were assessed as described previously in Martínez-López et al. (2014), with slight modification. Only GO terms which belong to the GO level 4, according to the function getLevel of the goProfiles R package (Sánchez et al., 2013), were considered.

## Author contributions

Nathalia M. V. Flórez-Zapata made substantial contributions to the design of the research, performed the experiments with the help of Fernando Hernandez-Godínez, performed the bioinformatics analyses, interpreted the results and wrote the first draft of the manuscript. M. H. Reyes-Valdés collaborated in the design of the research, suggested the protocol for male meiocytes isolation and checked its performance, provided plant materials, helped in the design of the experiments as well as in the interpretation of the results, making important intellectual contributions and critically reviewed the manuscript. Fernando Hernandez-Godínez modified and tested the protocol for meiocyte isolation, helped to cultivate plants, and assisted in the isolation of RNA and the construction of the libraries. Octavio Martínez conceived and designed the research, directed the bioinformatic and statistical analyses, contributed to the interpretation of the results and edited the manuscript. All authors have read the final version of the manuscript and agree with its content.

## Funding

This research was funded by grants to Octavio Martínez Conacyt project 165778 and Nathalia M. V. Flórez-Zapata Conacyt scholarship 262855.

### Conflict of interest statement

The authors declare that the research was conducted in the absence of any commercial or financial relationships that could be construed as a potential conflict of interest.
