# Supplementary material for: Transcriptomic landscape of prophase I sunflower male meiocytes
Source: Front Plant Sci. 2014 Jun 16;5:277. doi: 10.3389/fpls.2014.00277 (PMC4059168; doi:10.3389/fpls.2014.00277)
Supplement: Supplementary file 1 [file DataSheet1.PDF]

Frontiers in Plant Science

Research Article
28 May 2014

Supplementary Material: Transcriptomic landscape of prophase I sunflower male meiocytes

Nathalia M.V. Flórez-Zapata [1], M. Humberto Reyes-Valdés [2], Fernando Hernandez-Godínez[1] and Octavio Martínez [1,*]

[1] *Laboratorio Nacional de Genómica para la Biodiversidad (Langebio), Centro de Investigación y de Estudios Avanzados del Instituto Politécnico Nacional (Cinvestav), 36821, Irapuato, Guanajuato, México*
[2] *Department of Plant Breeding, Universidad Autónoma Agraria Antonio Narro, Calzada Antonio Narro 1293, C.P. 25315, Saltillo, Coahuila, México*

Correspondence*:
Octavio Martínez
Laboratorio Nacional de Genómica para la Biodiversidad (LANGEBIO), Centro de Investigación y de Estudios Avanzados del Instituto Politécnico Nacional (Cinvestav), 36821, Irapuato, Guanajuato, México,
omartine@langebio.cinvestav.mx

Plant Meiosis - Global approaches

1 SUPPLEMENTARY TABLES AND FIGURES

Supplementary Figures, referred to in the main text as “Figure S1” to “Figure S3” and Supplementary Tables, referred to in the main text as “Table S1” to “Table S10” are presented in this supplement.

		Green Plants																								
		Land Plants																								
		Vascular Plants																								
		Angiosperms																								
		Eudicots																								
		Fabids																								
		N2 - Fixing Clade								Malvids					Monocots							Chlorophyta				
		Papilionoideae					Malpighiales			Brassicales					BEP Clade			PACCMADClade				Memiellales				
		Galegoids			Rosaceae		Euphorbiaceae			Arabidopsis					Oryza							Ostreococcus			Chlamydomonadales	
**Locus**	**Symbol**	Lja	Mtr	Gma	Mdo	Fve	Mes	Rco	Ptr	Ath	Aly	Cpa	Tca	Vvi	Osa	Osain	Bdi	Sbi	Zma	Smo	Ppa	Olu	Ota	M. sp.	Vca	Cre
AT1G14750	SDS	1		1	2	1	1	1	1	1	1	1	1	1	1	2	1	1	2							
AT1G22275	ZYP1b		1	2		5	1	1	1	2	2	1	1	1	1	1	1	2	1	1						
AT1G64030	ATSRP3									1	1															
AT1G75950	ASK1	1	2	3	1	2	2	3	3	2	1	2	1	2	2	2	2	2	2	1	2			1	1	1
AT1G77390	CYCA1	1	1	4	2	1	2	1	2	1	1	1	1	1	1	2	2	3		2	8			1	1	1
AT2G14540	ATSRP2									1	1															
AT2G27170	SMC3	2	1	2	2	1	1	1	1	1	1	1	1	1	2	3	1	2	1			1	1	1	1	1
AT2G42890	AML2	1	1	4		2	2	2	2	3	3	2	2	3	2	2	2	1	2	1	3				1	2
AT3G19590	BUB3.1	1	1	3	2	1	1	1	2	2	2	1	1	1	1	1	1	1	1		1			1	1	1
AT3G29350	AHP2	1	4	4	2		2	2	3	2	2	2	2	2			2	1			3	1	1	1	1	1
AT3G47460	ATSMC2	2		2	4	3	1	1	2	2	1	1	1	1	1	1	1	1	1	1	1	1	1	1	2	1
AT3G57860	OSD1									1	1															
AT4G00020	BRCA2A	1	2	1	1	1	1	1	1	2	1	1	1	1	1	1	1	1	1			1	1	1	1	1
AT4G05190	ATK5	3	4	8	5	2	2	2	4	4	2	2	2	2	2	3	2	2	1	2	2	1	1	1	1	1
AT4G27170	SESA4									1		5														
AT5G05490	SYN1		1	1	1	1	1	1	1	1	1	1	1	1	1	1	2	1	1	1						
AT5G07660	SMC6A	1	1	1	1	1	1	1	3	2	2	3	1	1	1	2	1	1	1	1	1	1	1	1	1	1
AT5G15920	SMC5	2	4	1	1	1	1	1	1	1	1	1	1	1	1	2	1	1	1	1	1	1	1	1	1	1
AT5G40840	SYN2	2	1		1	1	1	1	1	1	1	1	1	1					1							
AT5G51330	SWI1		1	2	3	1	1	1	2	1	1	1	1	2	1	1	1	1	1	2	3					
AT5G54260	MRE11	2	1	1	2	1	1	1	1	1	1	1	1	1	2	2	2	1	1	1	1	1	1	1	1	1

Lja: Lotus japonicus, Mtr: Medicago truncatula, Gma: Glycine max, Mdo: Malus domestica, Fve: Fragaria vesca, Mes: Manihot esculenta, Rco: Ricinus communis, Ptr: Populus trichocarpa,
Ath: Arabidopsis thaliana, Aly: Arabidopsis lyrata, Cpa: Carica papaya, Tca: Theobroma cacao, Vvi: Vitis vinifera, Osa: Oryza sativa ssp. japonica, Osain: Oryza sativa ssp. indica, Bdi: Brachypodium distachyon,
Sbi: Sorghum bicolor, Zma: Zea mays, Smo: Selaginella moellendorffii, Ppa: Physcomitrella patens, Olu: Ostreococcus lucimarinus, Ota: Ostreococcus tauri, M. sp.: Micromonas sp. RCC299, Vca: Volvox carteri, Cre: Chlamydomonas reinhardtii.

Supplementary Figure 1. Conservation of the family of orthologs of meiotic genes not detected in sunflower. Numbers represent the members of each family of orthologs for each species (data processed from the Plaza database).

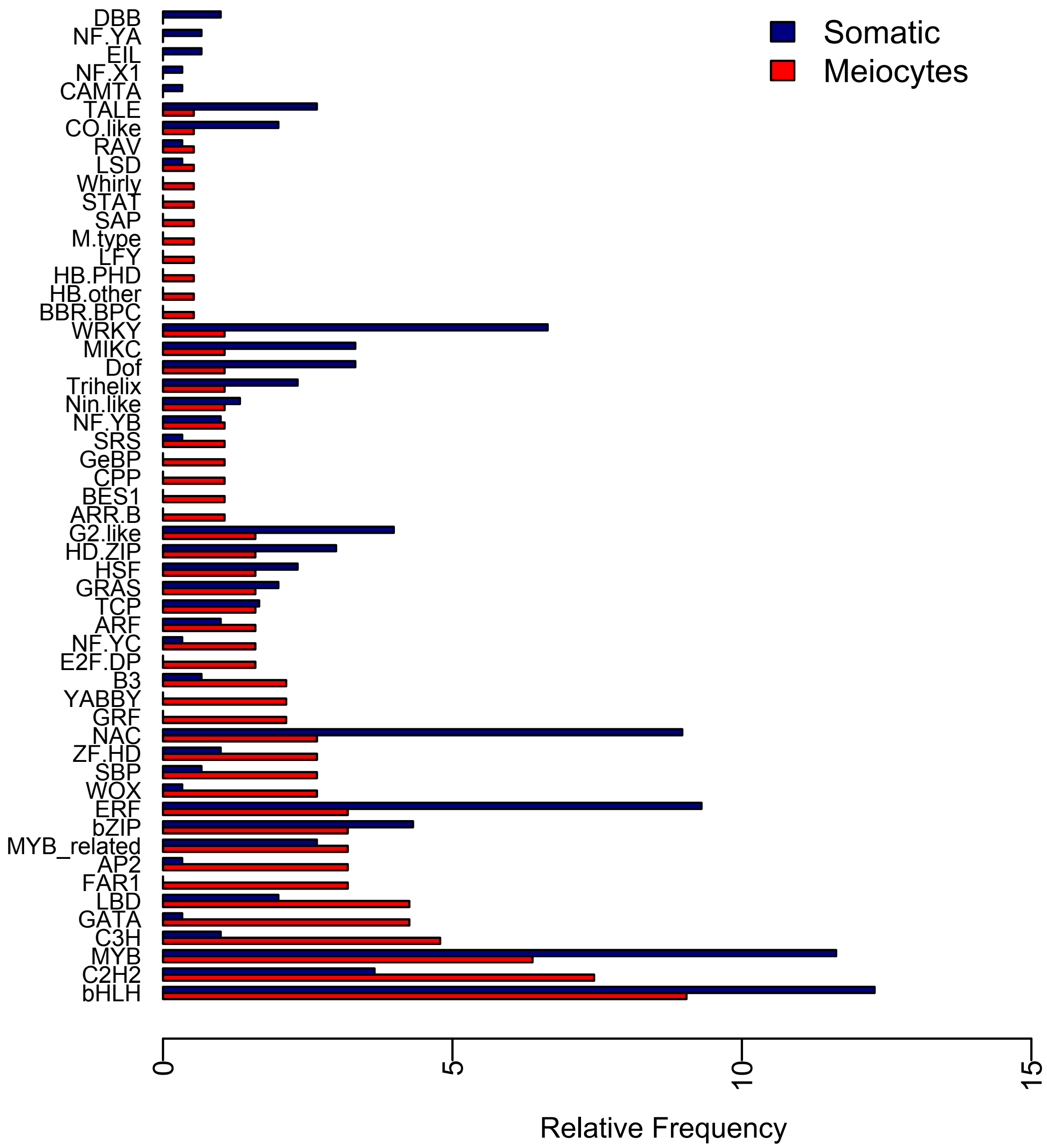

Supplementary Figure 2. Frequency of types of transcription factors (TF) found differentially expressed between meiocytes and somatic transcriptomes. The number of differentially expressed TF of each type are presented in relation of the total number of TF differentially expressed in each transcriptome.

Supplementary Table 1. Number of paired reads obtained (Total raw), quality trimmed (Trimmed) and uniquely mapped at each library. $BR1$ and $BR2$ are biological replicates of the meiocytes libraries. Percentage of the Trimmed are in reference to the Total raw while percentages of the Uniquely mapped are in reference to the Trimmed.

Library	Total raw	Trimmed	Uniquely mapped
Somatic	173,458,302	133,126,131 (76.75%)	100,607,075 (75.57%)
Meiocytes BR_1	175,789,421	139,227,278 (79.20%)	98,778,565 (70.95%)
Meiocytes BR_2	142,454,268	115,413,813 (81.02%)	79,611,917 (68.98%)
Total	491,701,991	387,767,222 (78.86%)	278,997,557 (71.94%)

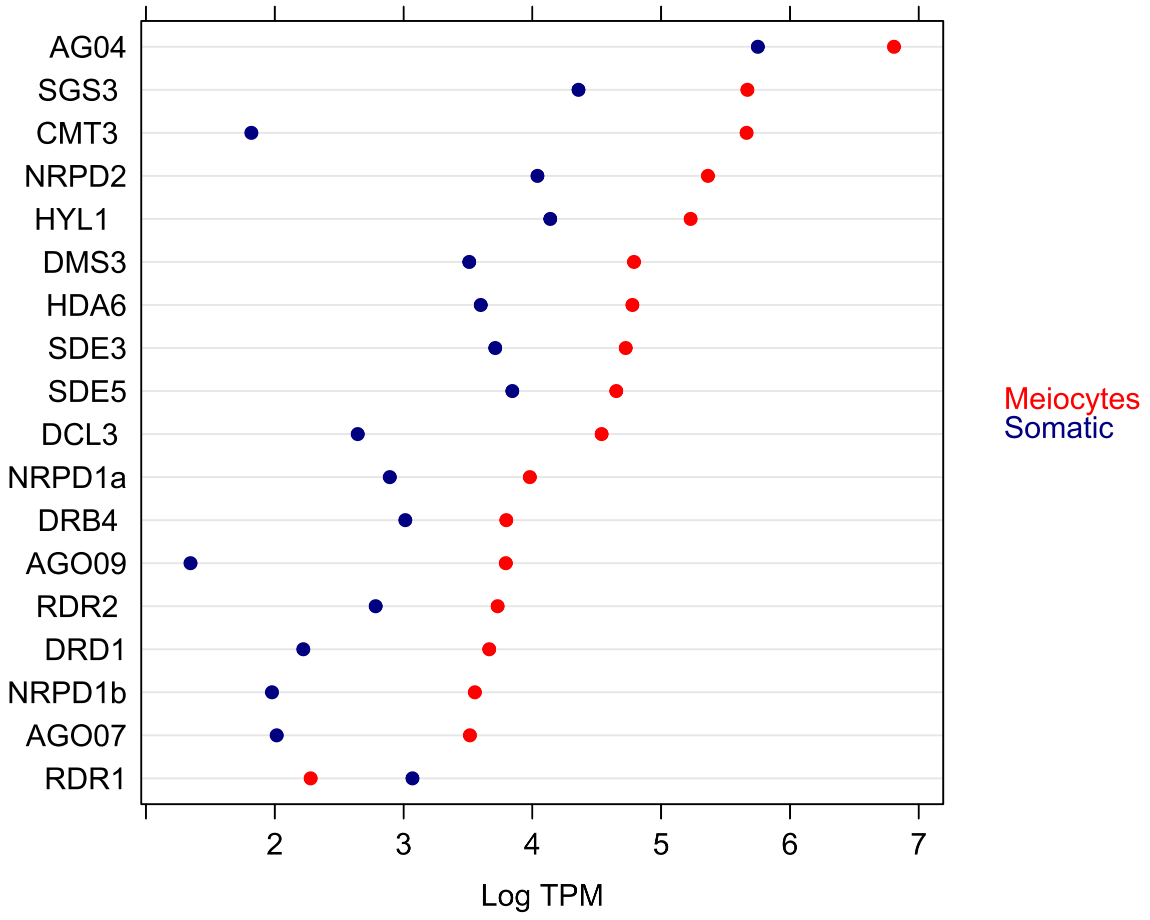

Supplementary Figure 3. Expression level (log of TPM) of differentially expressed (DE) genes with previously established function in gene silencing.

Supplementary Table 2. GO terms of CC categories with higher expression in the meiocytes library.

GO Id	GO Term definition	Fold Change
GO:0000796	condensin complex	32.70
GO:0000808	origin recognition complex	29.18
GO:0000776	kinetochore	25.38
GO:0042555	MCM complex	23.06
GO:0048196	middle lamella-containing extracellular matrix	16.10
GO:0032300	mismatch repair complex	12.41
GO:0044459	plasma membrane part	12.20
GO:0005971	ribonucleoside-diphosphate reductase complex	10.54
GO:0000811	GINS complex	7.37
GO:0000786	nucleosome	6.17
GO:0005663	DNA replication factor C complex	5.68
GO:0009331	glycerol-3-phosphate dehydrogenase complex	4.13
GO:0005640	nuclear outer membrane	3.93
GO:0033597	mitotic checkpoint complex	3.66
GO:0005678	chromatin assembly complex	3.58
GO:0033588	Elongator holoenzyme complex	3.57
GO:0048500	signal recognition particle	3.56
GO:0030880	RNA polymerase complex	3.52
GO:0010317	pyrophosphate-dependent phosphofructokinase complex, alpha-subunit complex	3.41
GO:0005731	nucleolus organizer region	3.00
GO:0005732	small nucleolar ribonucleoprotein complex	2.67
GO:0005658	alpha DNA polymerase:primase complex	2.67
GO:0008180	signalosome	2.60
GO:0005787	signal peptidase complex	2.55
GO:0005844	polysome	2.54
GO:0005838	proteasome regulatory particle	2.52
GO:0015934	large ribosomal subunit	2.51
GO:0005742	mitochondrial outer membrane translocase complex	2.44
GO:0005839	proteasome core complex	2.40
GO:0016272	prefoldin complex	2.33
GO:0019866	organelle inner membrane	2.30
GO:0035145	exon-exon junction complex	2.25
GO:0005875	microtubule associated complex	2.23
GO:0005758	mitochondrial intermembrane space	2.19
GO:0016591	DNA-directed RNA polymerase II, holoenzyme	2.18
GO:0009330	DNA topoisomerase complex (ATP-hydrolyzing)	2.16
GO:0005840	ribosome	2.15
GO:0000276	mitochondrial proton-transporting ATP synthase complex, coupling factor F(o)	2.07
GO:0005852	eukaryotic translation initiation factor 3 complex	2.05
GO:0005667	transcription factor complex	2.00

Supplementary Table 3. GO terms of CC categories with higher expression in the somatic library.

GO Id	GO Term definition	Fold Change
GO:0070825	micropyle	187.39
GO:0009782	photosystem I antenna complex	55.52
GO:0031012	extracellular matrix	54.90
GO:0012506	vesicle membrane	47.64
GO:0043674	columella	27.78
GO:0009512	cytochrome b6f complex	19.69
GO:0016328	lateral plasma membrane	16.93
GO:0009523	photosystem II	15.54
GO:0031226	intrinsic to plasma membrane	15.25
GO:0009522	photosystem I	14.46
GO:0009538	photosystem I reaction center	13.65
GO:0009654	oxygen evolving complex	13.64
GO:0005955	calcineurin complex	11.82
GO:0030076	light-harvesting complex	10.88
GO:0030118	clathrin coat	10.62
GO:0010598	NAD(P)H dehydrogenase complex (plastoquinone)	10.31
GO:0009544	chloroplast ATP synthase complex	9.77
GO:0009344	nitrite reductase complex [NAD(P)H]	9.40
GO:0009930	longitudinal side of cell surface	8.44
GO:0009783	photosystem II antenna complex	6.88
GO:0009517	PSII associated light-harvesting complex II	6.37
GO:0005945	6-phosphofructokinase complex	5.59
GO:0005754	mitochondrial proton-transporting ATP synthase, catalytic core	5.47
GO:0042170	plastid membrane	5.24
GO:0045259	proton-transporting ATP synthase complex	4.80
GO:0019898	extrinsic to membrane	4.17
GO:0031519	PcG protein complex	4.00
GO:0009579	thylakoid	3.79
GO:0009925	basal plasma membrane	3.77
GO:0045177	apical part of cell	3.18
GO:0009539	photosystem II reaction center	3.08
GO:0009986	cell surface	3.02
GO:0031224	intrinsic to membrane	3.01
GO:0009329	acetate CoA-transferase complex	3.01
GO:0031897	Tic complex	2.97
GO:0009898	internal side of plasma membrane	2.85
GO:0042175	nuclear outer membrane-endoplasmic reticulum membrane network	2.85
GO:0005615	extracellular space	2.83
GO:0031227	intrinsic to endoplasmic reticulum membrane	2.69
GO:0005835	fatty acid synthase complex	2.68
GO:0005618	cell wall	2.61
GO:0071944	cell periphery	2.37
GO:0010007	magnesium chelatase complex	2.33
GO:0043233	organelle lumen	2.33
GO:0090406	pollen tube	2.23
GO:0005960	glycine cleavage complex	2.22
GO:0090404	pollen tube tip	2.12
GO:0005625	soluble fraction	2.08
GO:0000813	ESCRT I complex	2.07
GO:0042651	thylakoid membrane	2.01

Supplementary Table 4. GO terms of BP categories with higher expression in the meiocytes library.

GO Id	GO Term definition	Fold Change
GO:0048658	tapetal layer development	133.97
GO:0010865	stipule development	108.00
GO:0045132	meiotic chromosome segregation	67.93
GO:0010069	zygote asymmetric cytokinesis in embryo sac	49.26
GO:0007108	cytokinesis, initiation of separation	35.38
GO:0051026	chiasma assembly	27.93
GO:0009856	pollination	26.72
GO:0030155	regulation of cell adhesion	24.58
GO:0051304	chromosome separation	17.09
GO:0045143	homologous chromosome segregation	15.75
GO:0007050	cell cycle arrest	15.07
GO:0048479	style development	14.08
GO:0000086	G2/M transition of mitotic cell cycle	14.05
GO:0048316	seed development	13.59
GO:0010529	negative regulation of transposition	13.55
GO:0010376	stomatal complex formation	11.46
GO:0010234	tapetal cell fate specification	11.18
GO:0051171	regulation of nitrogen compound metabolic process	9.85
GO:0019079	viral genome replication	9.62
GO:0007131	reciprocal meiotic recombination	7.50
GO:0010440	stomatal lineage progression	7.20
GO:0009755	hormone-mediated signaling pathway	6.97
GO:0010941	regulation of cell death	6.85
GO:0006311	meiotic gene conversion	6.27
GO:0010254	nectary development	6.08
GO:0009413	response to flooding	5.99
GO:0042138	meiotic DNA double-strand break formation	4.54
GO:0042754	negative regulation of circadian rhythm	4.21
GO:0002679	respiratory burst involved in defense response	4.08
GO:0022619	generative cell differentiation	4.06
GO:2000036	regulation of stem cell maintenance	4.06
GO:0010344	seed oilbody biogenesis	3.96
GO:0042127	regulation of cell proliferation	3.89
GO:0006072	glycerol-3-phosphate metabolic process	3.84
GO:0000819	sister chromatid segregation	3.78
GO:0010229	inflorescence development	3.64
GO:0048653	anther development	3.48
GO:0055047	generative cell mitosis	3.31
GO:0048281	inflorescence morphogenesis	3.29
GO:0009850	auxin metabolic process	3.26
GO:0044249	cellular biosynthetic process	3.26
GO:0010450	inflorescence meristem growth	3.24
GO:0009314	response to radiation	3.20
GO:0000706	meiotic DNA double-strand break processing	3.16
GO:0000082	G1/S transition of mitotic cell cycle	3.09
GO:0048638	regulation of developmental growth	3.01
GO:0044403	symbiosis, encompassing mutualism through parasitism	2.83
GO:0080157	regulation of plant-type cell wall organization or biogenesis	2.70
GO:0010358	leaf shaping	2.68
GO:0000710	meiotic mismatch repair	2.68
GO:0032204	regulation of telomere maintenance	2.68
GO:0000910	cytokinesis	2.66
GO:0048513	organ development	2.64
GO:0016197	endosome transport	2.63
GO:0000917	barrier septum assembly	2.59
GO:2000026	regulation of multicellular organismal development	2.55

Supplementary Table 4. GO terms of BP categories with higher expression in the meiocytes library (continuation).

GO Id	GO Term definition	Fold Change
GO:0007530	sex determination	2.55
GO:0045787	positive regulation of cell cycle	2.53
GO:0009799	specification of symmetry	2.52
GO:0006342	chromatin silencing	2.51
GO:0008356	asymmetric cell division	2.50
GO:0006446	regulation of translational initiation	2.49
GO:0010220	positive regulation of vernalization response	2.44
GO:0010081	regulation of inflorescence meristem growth	2.39
GO:0010022	meristem determinacy	2.39
GO:0035266	meristem growth	2.31
GO:0019048	virus-host interaction	2.27
GO:0051726	regulation of cell cycle	2.27
GO:0010015	root morphogenesis	2.27
GO:0048827	phyllome development	2.25
GO:0048577	negative regulation of short-day photoperiodism, flowering	2.16
GO:0050792	regulation of viral reproduction	2.15
GO:0048235	pollen sperm cell differentiation	2.14
GO:0060548	negative regulation of cell death	2.07
GO:0009652	thigmotropism	2.04
GO:0048528	post-embryonic root development	2.03
GO:0006793	phosphorus metabolic process	2.02
GO:0006897	endocytosis	2.01
GO:0031047	gene silencing by RNA	2.01

Supplementary Table 5. GO terms of BP categories with higher expression in the somatic library.

GO Id	GO Term definition	Fold Change
GO:0010647	positive regulation of cell communication	142.00
GO:0048838	release of seed from dormancy	138.69
GO:0080090	regulation of primary metabolic process	76.00
GO:0009835	ripening	74.73
GO:0010221	negative regulation of vernalization response	74.29
GO:0022603	regulation of anatomical structure morphogenesis	70.60
GO:0009606	tropism	47.81
GO:0015977	carbon fixation	30.69
GO:0010623	developmental programmed cell death	29.76
GO:0009830	cell wall modification involved in abscission	27.70
GO:0006066	alcohol metabolic process	19.28
GO:0048657	tapetal cell differentiation	17.84
GO:0080027	response to herbivore	17.47
GO:0006723	cuticle hydrocarbon biosynthetic process	14.50
GO:0045848	positive regulation of nitrogen utilization	14.00
GO:0034059	response to anoxia	13.15
GO:2000072	regulation of defense response to fungus, incompatible interaction	12.25
GO:0007584	response to nutrient	10.22
GO:0023014	signal transduction by phosphorylation	9.85
GO:0080086	stamen filament development	9.78
GO:0010241	ent-kaurene oxidation to kaurenoic acid	8.78
GO:0030397	membrane disassembly	8.56
GO:0060862	negative regulation of floral organ abscission	8.19
GO:0010942	positive regulation of cell death	8.19
GO:0006949	syncytium formation	7.96
GO:0009638	phototropism	7.91
GO:0010483	pollen tube reception	7.88
GO:0051211	anisotropic cell growth	7.77
GO:0080170	hydrogen peroxide transmembrane transport	7.67
GO:0050891	multicellular organismal water homeostasis	7.50
GO:2000068	regulation of defense response to insect	6.77
GO:0051090	regulation of sequence-specific DNA binding transcription factor activity	6.77
GO:0017145	stem cell division	6.35
GO:0015833	peptide transport	6.22
GO:0009629	response to gravity	5.99
GO:0010274	hydrotropism	5.94
GO:0048359	mucilage metabolic process involved seed coat development	5.87
GO:0048544	recognition of pollen	5.65
GO:0015931	nucleobase-containing compound transport	5.53
GO:2000652	regulation of secondary cell wall biogenesis	5.48
GO:0015979	photosynthesis	5.45
GO:0001666	response to hypoxia	5.39
GO:0045926	negative regulation of growth	5.39
GO:0019419	sulfate reduction	5.26
GO:0010431	seed maturation	5.21
GO:0048826	cotyledon morphogenesis	5.21
GO:0048438	floral whorl development	5.04
GO:1900056	negative regulation of leaf senescence	4.97
GO:0048444	floral organ morphogenesis	4.83
GO:0080126	ovary septum development	4.72
GO:0051607	defense response to virus	4.58
GO:0010618	aerenchyma formation	4.41
GO:0009864	induced systemic resistance, jasmonic acid mediated signaling pathway	4.40
GO:0009414	response to water deprivation	4.33
GO:0010260	organ senescence	4.11
GO:0009866	induced systemic resistance, ethylene mediated signaling pathway	3.95
GO:0009268	response to pH	3.68
GO:0006855	drug transmembrane transport	3.64

Supplementary Table 5. GO terms of BP categories with higher expression in the somatic library (continuation).

GO Id	GO Term definition	Fold Change
GO:0010256	endomembrane system organization	3.58
GO:0048480	stigma development	3.45
GO:0031349	positive regulation of defense response	3.43
GO:0009612	response to mechanical stimulus	3.26
GO:0009620	response to fungus	3.18
GO:0008610	lipid biosynthetic process	3.17
GO:0006792	regulation of sulfur utilization	3.05
GO:0009625	response to insect	3.02
GO:0007389	pattern specification process	2.96
GO:0072593	reactive oxygen species metabolic process	2.95
GO:0046283	anthocyanin metabolic process	2.86
GO:0080167	response to karrikin	2.77
GO:0009624	response to nematode	2.67
GO:0060148	positive regulation of posttranscriptional gene silencing	2.64
GO:0019538	protein metabolic process	2.60
GO:0005976	polysaccharide metabolic process	2.60
GO:0009698	phenylpropanoid metabolic process	2.60
GO:0045165	cell fate commitment	2.51
GO:0006644	phospholipid metabolic process	2.49
GO:0030036	actin cytoskeleton organization	2.44
GO:0001887	selenium compound metabolic process	2.43
GO:0006730	one-carbon metabolic process	2.43
GO:0009636	response to toxin	2.40
GO:0010017	red or far-red light signaling pathway	2.32
GO:0009825	multidimensional cell growth	2.32
GO:0010617	circadian regulation of calcium ion oscillation	2.32
GO:0051410	detoxification of nitrogen compound	2.30
GO:0048317	seed morphogenesis	2.28
GO:0009611	response to wounding	2.27
GO:0051707	response to other organism	2.26
GO:0016998	cell wall macromolecule catabolic process	2.26
GO:0010112	regulation of systemic acquired resistance	2.26
GO:0032922	circadian regulation of gene expression	2.25
GO:0032940	secretion by cell	2.24
GO:0009308	amine metabolic process	2.20
GO:0016051	carbohydrate biosynthetic process	2.20
GO:0045927	positive regulation of growth	2.17
GO:0045595	regulation of cell differentiation	2.17
GO:0007267	cell-cell signaling	2.15
GO:0009653	anatomical structure morphogenesis	2.14
GO:0007009	plasma membrane organization	2.13
GO:0006629	lipid metabolic process	2.12
GO:0006952	defense response	2.12
GO:0008104	protein localization	2.10
GO:0048833	specification of floral organ number	2.02
GO:0009266	response to temperature stimulus	2.01

Supplementary Table 6. GO terms of MF categories with higher expression in the meiocytes library.

GO Id	GO Term definition	Fold Change
GO:0050662	coenzyme binding	6.01
GO:0016018	cyclosporin A binding	4.15
GO:0090353	polygalacturonase inhibitor activity	4.13
GO:0016830	carbon-carbon lyase activity	3.73
GO:0042393	histone binding	3.04
GO:0030332	cyclin binding	2.98
GO:0031491	nucleosome binding	2.60
GO:0001671	ATPase activator activity	2.10

Supplementary Table 7. GO terms of MF categories with higher expression in the somatic library.

GO Id	GO Term definition	Fold Change
GO:0051738	xanthophyll binding	279.04
GO:0051213	dioxygenase activity	180.30
GO:0008379	thioredoxin peroxidase activity	106.29
GO:0080015	sabinene synthase activity	97.62
GO:0046863	ribulose-1,5-bisphosphate carboxylase/oxygenase activator activity	91.16
GO:0010313	phytochrome binding	30.59
GO:0080016	(-)-E-beta-caryophyllene synthase activity	18.31
GO:0080017	alpha-humulene synthase activity	18.31
GO:0010242	oxygen evolving activity	14.63
GO:0045435	lycopene epsilon cyclase activity	12.12
GO:0016168	chlorophyll binding	11.52
GO:0016701	oxidoreductase activity, acting on single donors with incorporation of molecular oxygen	9.77
GO:0000155	two-component sensor activity	9.60
GO:0044183	protein binding involved in protein folding	7.50
GO:0051740	ethylene binding	7.13
GO:0000170	sphingosine hydroxylase activity	6.96
GO:2001147	camalexin binding	6.43
GO:2001227	quercitrin binding	6.43
GO:0016798	hydrolase activity, acting on glycosyl bonds	6.37
GO:0008559	xenobiotic-transporting ATPase activity	6.35
GO:0004602	glutathione peroxidase activity	6.23
GO:0015197	peptide transporter activity	6.22
GO:0030674	protein binding, bridging	5.90
GO:0004096	catalase activity	5.49
GO:0008517	folic acid transporter activity	5.34
GO:0043495	protein anchor	5.32
GO:0000900	translation repressor activity, nucleic acid binding	5.04
GO:0008428	ribonuclease inhibitor activity	4.83
GO:0005529	sugar binding	4.54
GO:0051861	glycolipid binding	4.33
GO:0045436	lycopene beta cyclase activity	4.30
GO:0045550	geranylgeranyl reductase activity	4.05
GO:0046422	violaxanthin de-epoxidase activity	3.97
GO:0005344	oxygen transporter activity	3.78
GO:0008398	sterol 14-demethylase activity	3.75
GO:0046423	allene-oxide cyclase activity	3.53
GO:0015238	drug transmembrane transporter activity	3.50
GO:0005522	profilin binding	3.34
GO:0046910	pectinesterase inhibitor activity	3.27
GO:0071992	phytochelatin transmembrane transporter activity	2.91
GO:0031210	phosphatidylcholine binding	2.90
GO:0004134	4-alpha-glucanotransferase activity	2.87
GO:0016866	intramolecular transferase activity	2.79
GO:0017077	oxidative phosphorylation uncoupler activity	2.67
GO:0016846	carbon-sulfur lyase activity	2.66
GO:0080132	fatty acid alpha-hydroxylase activity	2.53
GO:0005496	steroid binding	2.50
GO:0042802	identical protein binding	2.40
GO:0016667	oxidoreductase activity, acting on a sulfur group of donors	2.35
GO:0010331	gibberellin binding	2.35
GO:0004888	transmembrane signaling receptor activity	2.33
GO:0016788	hydrolase activity, acting on ester bonds	2.24
GO:0030247	polysaccharide binding	2.20
GO:0016757	transferase activity, transferring glycosyl groups	2.15
GO:0003876	AMP deaminase activity	2.11
GO:0005516	calmodulin binding	2.08
GO:0005543	phospholipid binding	2.06
GO:0031418	L-ascorbic acid binding	2.05
GO:0030170	pyridoxal phosphate binding	2.02

Supplementary Table 8. Metabolic pathways with higher expression in the meiocytes library.

Pathways	Fold Change
sporopollenin precursor biosynthesis	360.44
anthocyanin biosynthesis (pelargonidin 3-O-glucoside, cyanidin 3-O-glucoside)	42.61
superpathway of anthocyanin biosynthesis (from cyanidin and cyanidin 3-O-glucoside)	39.90
IAA degradation IV	12.70
indole-3-acetyl-amino acid biosynthesis	12.70
alpha-amyrin biosynthesis	11.46
proanthocyanidin biosynthesis from flavanols	10.17
lysine degradation I	9.62
galactose degradation I (Leloir pathway)	5.07
pyrimidine deoxyribonucleotides de novo biosynthesis	3.57
glycerol-3-phosphate shuttle	2.99
cutin biosynthesis	2.77
guanine and guanosine salvage II	2.66
ubiquinone-9 biosynthesis (eukaryotic)	2.44
PRPP biosynthesis I	2.34
1,4-dihydroxy-2-naphthoate biosynthesis II (plants)	2.32
glutamate degradation I	2.24
hydroxycinnamic acid tyramine amides biosynthesis	2.10
methionine degradation II	2.09
oxidative ethanol degradation I	2.08
alanine biosynthesis III	2.06
ferulate and sinapate biosynthesis	2.02

Supplementary Table 9. Metabolic pathways with higher expression in the somatic library.

Pathways	Fold Change
lanosterol biosynthesis	89.20
nicotianamine biosynthesis	84.67
fatty acid alpha-oxidation	73.36
xanthophyll cycle	68.70
antheraxanthin and violaxanthin biosynthesis	68.70
arginine degradation VI (arginase 2 pathway)	64.82
camalexin biosynthesis	64.00
farnesene biosynthesis	55.50
pyruvate fermentation to ethanol II	47.76
acetaldehyde biosynthesis I	47.76
sinapate ester biosynthesis	40.77
ent-kaurene biosynthesis	28.93
zeaxanthin biosynthesis	26.10
methylquercetin biosynthesis	26.01
homogalacturonan degradation	25.27
nitrate reduction II (assimilatory)	23.52
gibberellin inactivation	18.41
beta-caryophyllene biosynthesis	18.31
phaseic acid biosynthesis	17.38
abscisic acid glucose ester biosynthesis	16.32
asparagine biosynthesis I	16.13
photosynthesis light reactions	15.89
UDP-D-galacturonate biosynthesis I (from UDP-D-glucuronate)	14.70
2,4,6-trinitrotoluene degradation	14.55
(E,E)-4,8,12-trimethyltrideca-1,3,7,11-tetraene biosynthesis	14.34
(3E)-4,8-dimethylnona-1,3,7-triene biosynthesis	14.12
ethanol degradation	14.10
lutein biosynthesis	14.00
monoterpene biosynthesis	13.83
UDP-glucose biosynthesis (from sucrose)	12.97
delta-carotene biosynthesis	12.12
monolignol glucosides biosynthesis	11.35
coniferin metabolism	11.35
salicylate glucosides biosynthesis IV	10.81
putrescine biosynthesis IV	10.58
putrescine biosynthesis I	10.58
cytokinins-O-glucoside biosynthesis	9.74
hydroxyjasmonate sulfate biosynthesis	9.65
indole glucosinolate breakdown (active in intact plant cell)	9.38
melibiose degradation	8.86
GA12 biosynthesis	8.79
glutamate degradation IV	8.71
phytol salvage pathway	8.43
phospholipid desaturation	8.43
glucosinolate biosynthesis from phenylalanine	8.22
glucosinolate biosynthesis from tryptophan	8.15
13-LOX and 13-HPL pathway	8.12
trans-lycopene biosynthesis II (plants)	8.09
glucosinolate biosynthesis from pentahomomethionine	7.75
glucosinolate biosynthesis from hexahomomethionine	7.75
poly-hydroxy fatty acids biosynthesis	7.66
glutamate biosynthesis IV	7.66
ajugose biosynthesis II (galactinol-independent)	7.57
galactosylcyclitol biosynthesis	7.57
thiamine biosynthesis II	7.41
quercetinsulphates biosynthesis	7.29

Supplementary Table 9. Metabolic pathways with higher expression in the somatic library (continuation 1).

Pathways	Fold Change
methyl indole-3-acetate interconversion	7.05
citrulline biosynthesis	6.90
cyanide degradation	6.53
cytokinins degradation	6.51
sorbitol degradation I	6.42
ammonia assimilation cycle II	6.33
stachyose biosynthesis	6.21
glutathione redox reactions I	6.10
mannitol degradation II	6.10
UDP-D-glucuronate biosynthesis (from myo-inositol)	6.05
jasmonoyl-amino acid conjugates biosynthesis I	5.79
glutamine biosynthesis I	5.78
sulfate activation for sulfonation	5.40
ribose degradation	5.33
serine biosynthesis	5.32
oxidized GTP and dGTP detoxification	5.31
glucosinolate biosynthesis from tetrahomomethionine	5.23
glucosinolate biosynthesis from trihomomethionine	5.23
glucosinolate biosynthesis from homomethionine	5.23
Fe(III)-reduction and Fe(II) transport	5.10
gibberellin biosynthesis I (non C-3, non C-13 hydroxylation)	5.06
gibberellin biosynthesis II (early C-3 hydroxylation)	5.06
gibberellin biosynthesis III (early C-13 hydroxylation)	5.06
alanine degradation III	5.04
alanine biosynthesis II	5.04
ajugose biosynthesis I (galactinol-dependent)	4.96
vitamin E biosynthesis	4.92
nonaprenyl diphosphate biosynthesis II	4.85
linoleate biosynthesis I (plants)	4.79
sulfate reduction II (assimilatory)	4.76
flavonoid biosynthesis	4.73
phenylpropanoid biosynthesis, initial reactions	4.65
trans-cinnamoyl-CoA biosynthesis	4.63
benzoate biosynthesis II (CoA-independent, non-β-oxidative)	4.63
cellulose biosynthesis	4.46
photorespiration	4.43
ethylene biosynthesis from methionine	4.31
beta-carotene biosynthesis	4.30
urea cycle	4.18
cuticular wax biosynthesis	4.11
phytyl diphosphate biosynthesis	4.05
uracil degradation (reductive)	3.86
choline biosynthesis I	3.80
pentose phosphate pathway (non-oxidative branch)	3.78
proline biosynthesis III	3.74
free phenylpropanoid acid biosynthesis	3.70
phenylethanol biosynthesis	3.66
nonaprenyl diphosphate biosynthesis III	3.66
geranylgeranyldiphosphate biosynthesis	3.66
methylerythritol phosphate pathway	3.60
chlorophyll a degradation	3.54
mannose degradation	3.49
aldehyde oxidation I	3.39
chlorophyll a biosynthesis II	3.39
glucosinolate biosynthesis from dihomomethionine	3.36
glycolipid desaturation	3.35

Supplementary Table 9. Metabolic pathways with higher expression in the somatic library (continuation 2).

Pathways	Fold Change
trans-zeatin biosynthesis	3.30
simple coumarins biosynthesis	3.24
aspartate biosynthesis	3.24
galactose degradation III	3.19
phytate degradation II	3.15
Calvin-Benson-Bassham cycle	3.10
UDP-L-rhamnose biosynthesis	3.03
chlorophyllide a biosynthesis I	3.02
phospholipases	2.99
cytokinins 7-N-glucoside biosynthesis	2.93
cytokinins 9-N-glucoside biosynthesis	2.93
glycine betaine biosynthesis III (plants)	2.92
triacylglycerol degradation	2.90
jasmonic acid biosynthesis	2.89
UDP-L-arabinose biosynthesis I (from UDP-xylose)	2.88
siroheme biosynthesis	2.87
homomethionine biosynthesis	2.83
choline biosynthesis III	2.83
lupeol biosynthesis	2.81
S-adenosylmethionine biosynthesis	2.76
pentose phosphate pathway (oxidative branch)	2.67
pyridine nucleotide cycling (plants)	2.64
cyanide detoxification	2.54
folate polyglutamylation II	2.52
UDP-D-xylose biosynthesis	2.51
farnesylcysteine salvage pathway	2.51
cysteine biosynthesis I	2.47
oleate biosynthesis I (plants)	2.47
S-adenosyl-L-methionine cycle II	2.46
aliphatic glucosinolate biosynthesis, side chain elongation cycle	2.43
brassinosteroid biosynthesis III	2.41
sulfite oxidation IV	2.41
glutathione degradation	2.40
sucrose biosynthesis	2.38
xyloglucan biosynthesis	2.36
4-hydroxyphenylpyruvate biosynthesis	2.35
chlorophyll cycle	2.31
plastoquinone-9 biosynthesis	2.28
GDP-L-fucose biosynthesis II (from L-fucose)	2.26
beta-alanine biosynthesis I	2.25
leucine degradation I	2.24
quercetin glucoside biosynthesis (Arabidopsis)	2.23
sphingolipid biosynthesis (plants)	2.22
pyruvate fermentation to lactate	2.18
very long chain fatty acid biosynthesis	2.13
dTDP-L-rhamnose biosynthesis II	2.12
gluconeogenesis	2.12
sucrose degradation III	2.12
1D-myo-inositol hexakisphosphate biosynthesis III (Spirodela polyrrhiza)	2.10
glycolysis I (plastidic)	2.10
serine racemization	2.07
UDP-D-apiose biosynthesis (from UDP-D-glucuronate)	2.06
brassinosteroid biosynthesis I	2.06
myo-inositol biosynthesis	2.04
methionine salvage pathway	2.04

Supplementary Table 10. Transcription factors exclusively expressed in meiocytes.

Ortholog (TAIR id)	Expression in Meiocytes (TPM)	Type
AT2G16910	214.62	bHLH
AT1G18960	97.90	MYB related
AT2G17950	9.52	WOX
AT3G12680	6.22	C3H
AT1G75430	4.25	TALE
AT3G04070	2.38	NAC
AT5G53210	2.00	bHLH
AT2G45410	1.89	LBD
AT1G06280	1.33	LBD
AT1G18790	1.11	Nin-like
AT3G13890	0.58	MYB
AT2G28810	0.42	Dof
AT3G18010	0.38	WOX
AT1G51120	0.17	RAV

Supplementary Table 11. Genes related to RNA-regulated gene silencing pathways.

Symbol	Ortholog (TAIR id)	Description
AGO01	AT1G48410	RNA slicer/core component of plant RISC
AGO02	AT1G31280	RNA slicer/involved in antiviral defense response
AGO03	AT1G31290	RNA slicer
AGO04	AT2G27040	RNA slicer/involved in the establishment phase of RdDM
AGO05	AT2G27880	RNA slicer
AGO06	AT2G32940	RNA slicer/rasiRNA-directed heterochromatin formation
AGO07	AT1G69440	RNA slicer/tasiRNA biogenesis and juvenile-to-adult transition
AGO08	AT5G21030	RNA slicer
AGO09	AT5G21150	RNA slicer/silencing the activity of TEs activity in the female gametophyte
AGO10	AT5G43810	RNA slicer
CMT3	AT1G69770	Methyltransferase/maintenance phase of RdDM
DCL1	AT1G01040	RNase III/miRNA, natsiRNA, and tasiRNA biogenesis
DCL2	AT3G03300	RNase III/natsiRNA biogenesis and viral defense
DCL3	AT3G43920	RNase III/rasiRNA biogenesis and establishment phase of RdDM
DCL4	AT5G20320	RNase III/tasiRNA biogenesis and viral defense
DRM2	AT3G17310	Methyltransferase/establishment phase of RdDM
HYL1	AT1G09700	dsRBP/miRNA and tasiRNA biogenesis
DRB4	AT3G62800	dsRBP/miRNA and tasiRNA biogenesis
DRD1	AT2G16390	SNF2-like chromatin-remodeling factor/establishment phase of RdDM
HEN1	AT4G29160	sRNA-specific methyltransferase/sRNA biogenesis
HST	AT3G05040	Exportin-5 ortholog/miRNA exportation from nucleus
MET1	AT5G49160	Methyltransferase/maintenance phase of RdDM methylation
NRPD1a	AT1G63020	DNA-dependent RNA polymerase/establishment phase of RdDM
NRPD1b	AT2G40030	DNA-dependent RNA polymerase/establishment phase of RdDM
NRPD2	AT3G23780	DNA-dependent RNA polymerase/establishment phase of RdDM
RDR1	AT1G14790	RNA-dependent RNA polymerase/viral defense
RDR2	AT4G11130	RNA-dependent RNA polymerase/rasiRNA biogenesis
RDR6	AT3G49500	RNA-dependent RNA polymerase/tasiRNA and natsiRNA biogenesis
SGS3	AT5G23570	Coiled-coil protein/tasiRNA and natsiRNA biogenesis
SDE3	AT1G05460	Silencing defective/Encodes a protein with similarity to RNA helicases
SDE5	AT3G15390	Similar to PRL1 interacting factor and is involved in virus induced silencing
DDM1	AT5G66750	Similar to SWI2/SNF2 chromatin remodeling proteins
DRD2	AT3G23780	Chromatin-remodeling factor
DMS3	AT3G49250	Involving secondary siRNA and spreading of DNA methylation
DMS4	AT2G30280	Transcriptional regulator functioning in RNA-directed DNA methylation and plant development
HDA6	AT5G63110	RPD3-like histone deacetylase/RNA-Mediated transcriptional silencing
MET1	AT5G49160	Encodes a cytosine methyltransferase
MOM1	AT1G08060	Encodes a transcriptional silencer/maintenance of methylation
